# AI-Generated Images of Substance Use and Recovery: Mixed Methods Case Study

**DOI:** 10.2196/81977

**Published:** 2026-02-19

**Authors:** Kathryn Heley, Jeffrey K Hom, Linnea Laestadius

**Affiliations:** 1 Zilber College of Public Health, University of Wisconsin Milwaukee, 1240 N 10th St, Milwaukee, WI, 53205, United States, 1 (414) 251-5607; 2 Rutgers Institute for Nicotine & Tobacco Studies, Rutgers University, New Brunswick, NJ, United States; 3 DrPH Program, Johns Hopkins Bloomberg School of Public Health, Baltimore, MD, United States; 4 Behavioral Health Services, San Francisco Department of Public Health, San Francisco, CA, United States

**Keywords:** Substance use, health communication, visual communication, artificial intelligence, generative AI, AI bias, implicit bias, stigma

## Abstract

**Background:**

Images created with generative artificial intelligence (AI) tools are increasingly used for health communication due to their ease of use, speed, accessibility, and low cost. However, AI-generated images may bring practical and ethical risks to health practitioners and the public, including through the perpetuation of stigma against vulnerable and historically marginalized groups.

**Objective:**

To understand the potential value of AI-generated images for health care and public health communication, we sought to analyze images of substance use disorder and recovery generated with ChatGPT. Specifically, we sought to investigate: (1) the default visual outputs produced in response to a range of prompts about substance use disorder and recovery, and (2) the extent to which prompt modification and guideline-informed prompting could mitigate potentially stigmatizing imagery.

**Methods:**

We performed a mixed-methods case study examining depictions of substance use and recovery in images generated by ChatGPT 4.o. We generated images (n=84) using (1) prompts with colloquial and stigmatizing language, (2) prompts that follow best practices for person-first language, (3) image prompts written by ChatGPT, and (4) a custom GPT informed by guidelines for images of SUD. We then used a mixed-methods approach to analyze images for demographics and stigmatizing elements.

**Results:**

Images produced in the default ChatGPT model featured primarily White men (81%, n=34). Further, images tended to be stigmatizing, featuring injection drug use, dark colors, and symbolic elements such as chains. These trends persisted even when person-first language prompts were used. Images produced by the guideline-informed custom GPT were markedly less stigmatizing; however, they featured almost only Black women (74%, n=31).

**Conclusions:**

Our findings confirm prior research about stigma and biases in AI-generated images and extend this literature to substance use. However, our findings also suggest that (1) images can be improved when clear guidelines are provided and (2) even with guidelines, iteration is needed to create an image that fully concords with best practices.

## Introduction

Generative artificial intelligence (AI) tools are now widely available and increasingly used for image generation. News outlets and public health entities have begun using text-to-image AI tools due to their ease of use, speed, accessibility, and low cost [1,2]. There have been calls for increased use of generative AI tools in public health, and AI has been described as critical to achieving “Public Health 3.0,” a public health practice that centers on cross-sector collaboration and the adoption of new skills, tools, and types of data to “meet the evolving challenges to population health.” [3] AI-generated images may also be promising for patient and medical education, including the creation of visual representations of patient narratives, illustrations for didactic lectures for medical students, and visual aids for patients [4].

AI-generated images also bring significant risks for health communication [5]. These images may perpetuate stigma, as AI platforms can be trained on biased datasets that reflect harmful stereotypes [6-9]. However, research to date has only examined images resulting from simple prompts, rather than from detailed parameters for appropriate images. Questions remain about AI-generated images in clinical training and health promotion, particularly for highly stigmatized topics.

In this study, we analyze ChatGPT-generated images of substance use disorder (SUD) and recovery. This focus is warranted and timely, as the media often uses images in its reporting of the country’s overdose crisis, and drug-related stigma is an impediment to care [10]. High levels of stigma exist toward people with SUDs among health care providers, whose preclinical education often incorporates ample images [11-14]. While no study has examined AI-generated images of substance use, prior research suggests that AI-generated images can reinforce mental health-related stigma by reflecting “historical biases and visual archetypes”.[6] Thus, AI-generated images may compound existing stigmas, raising ethical and practical concerns about the increased adoption of such images in clinical and public health communication materials.

Substance use also serves as a valuable case study, given existing literature on stigma reduction in health communication. Guidelines for empathic drug-related images have been developed, largely shaped by input from people with lived experience, while experimental research finds that depictions of recovery can reduce SUD-related stigma [15-20]. Additionally, research supports the use of “person-first” language and destigmatizing terms when describing SUD [21,22]. With the growth of text-to-image AI tools, it is critical to consider how the language used in prompts may impact resulting images.

To explore the content and implications of AI-generated images of substance use and recovery, we used a mixed-methods case study approach to analyze AI-generated images from ChatGPT, using commonly used terms and person-first language as prompts and varying the inclusion of detailed, empathy-oriented image guidelines. Findings also contribute to the understanding of the potential value of AI-generated images for health care and public health communication more broadly.

## Methods

We used a mixed-methods case study design to examine the outputs of a single AI model under different prompting conditions. A mixed-methods case study is appropriate when the objective is to understand a system in depth and to integrate qualitative and quantitative evidence to generate contextualized knowledge rather than to determine statistical generalization [23,24]. This approach is well-suited for exploratory work on emerging technologies and complex interactions between people and technology. Qualitative analysis allowed us to identify and interpret visual patterns, while quantitative coding helped formalize and organize the description of those patterns, strengthening internal validity and making our interpretation more systematic and comparable across prompts [25]. We selected this design because our goal was to characterize image outputs and examine how prompting strategies shape representational patterns, rather than to infer population-level effects or causal relationships.

### Model Selection

ChatGPT-4.o’s image model was launched in March 2025 (OpenAI). It is widely available and easy to access for public health professionals and the public, making it a relevant platform for exploring how SUD and recovery are represented in AI-generated imagery.

### Image Generation

Image generation followed a stepwise protocol to investigate: (1) the default visual outputs produced in response to different prompts about SUD and recovery; (2) the extent to which prompt modification and guideline-informed prompting could mitigate potentially stigmatizing imagery. Image generation was conducted by three researchers using ChatGPT Plus accounts, in three US states (ie, California, Maryland, Wisconsin) in June 2025. To remove bias and influence from prior interactions, chat history and memory were disabled, and each prompt was entered into a new chat session.

The format of each prompt was “Please make an image of [term or phrase].” If ChatGPT responded with suggestions, we replied, “Yes, please make an image that meets these criteria.” Major prompt categories included (see MultimediaAppendices 1  2 for all 14 prompts):

1. General terms (eg, “substance use disorder”), including some terms known to be stigmatizing (eg, “an addict”) because they are familiar to the public and continue to be used in health messaging despite guidelines [21,26]
2. Person-first language (eg, “a person with a substance use disorder”)
3. ChatGPT-written prompts aligned with best practices for SUD-related messaging (eg, “Please write a detailed prompt for a respectful and compassionate image of a person with a substance use disorder”). Resulting prompts were used to generate images

We then created a custom GPT—a tailored version of ChatGPT that incorporated additional knowledge. We uploaded five existing SUD-related image guidelines, with instructions for ChatGPT to adhere to these when creating images [15-19]. We then repeated all 14 prompts within this custom GPT.

### Coding and Analysis

Resulting images (n=84) were evaluated using a mixed-methods approach. In line with existing AI-generated image research, we first conducted a qualitative analysis to inductively identify recurring patterns. The research team conducted open-coding, identifying image features that aligned with the study aims. We separately reviewed all images, meeting regularly to review findings, resolve discrepancies, and strengthen confirmability and credibility.

To understand the frequency of image features, a structured coding instrument was created. Coding assessed demographics of the central figure in the image; visual features related to SUD and recovery; and the presence of stigmatizing and humanizing elements. Codes for the last of these were informed by the guideline documents on reducing SUD-related stigma in visual media and images. Codes for visual features and stigmatizing and humanizing elements were not mutually exclusive. Stigma was assessed based on the presence of six features synthesized from guidelines that were consistently recommended to be avoided to create “thoughtful representation” and “visuals that promote dignity, inclusion, and recovery”[15-19]. Specifically, these features were (1) showing paraphernalia, drugs, or active use, (2) dark setting, (3) dramatized, visualizing “rock bottom,” (4) visibly struggling, (5) isolation and internal sense of shame, and (6) messiness and chaos. If an image included at least 3 criteria from this list, it was labeled as a “yes” for “highly stigmatizing.” If an image included 1‐2 criteria from this list, it was labeled as “potentially stigmatizing.” If an image included no criteria, it was labeled as “no” for stigmatizing. All images were coded independently by two researchers using Microsoft Excel. Codes met conventional standards for adequate reliability, with high percent agreement and kappa values of 0.69 or higher [27]. Discrepancies were reconciled through discussion involving the third researcher. STATA (v.18.0; StataCorp) was used for all analyses. Through discussion, quantitative and qualitative data were refined into themes describing visual patterns across prompt types.

### Ethical Considerations

Approval by an institutional review board was not sought as the study did not involve human participants’ data.

## Results

### Overall Patterns in Images

Images generated by AI in response to SUD–related prompts revealed persistent stigmatizing patterns and limited representational diversity, with observable differences based on language used in the prompts, recovery framing, and the addition of guidelines. All investigators obtained similar images to the same prompts (MultimediaAppendices 1  2). Findings are organized by guideline use and then according to the major finding. See Table 1 for summary statistics of image content with and without guidelines.

**Table 1.** Description of AI-generated images without and with guidelines.

Code	Without guidelines (n=42), n (%)	With guidelines (n=42), n (%)
Number of persons		
None	1 (2)	0 (0)
One	41 (98)	6 (14)
Two or more	0 (0)	36 (86)
Race/ethnicity (central figure)		
White	36 (86)	5 (12)
Black	2 (5)	35 (83)
Hispanic	2 (5)	0 (0)
Asian	0 (0)	0 (0)
Unclear	1 (2)	2 (5)
No person	1 (2)	0 (0)
Gender (central figure)		
Woman	4 (10)	33 (79)
Man	37 (88)	8 (19)
Unclear	0 (0)	1 (2)
No person	1 (2)	0 (0)
Recovery Signifier^a^		
Chip/token/medallion	7 (17)	0 (0)
Other recovery signifier	14 (33)	6 (14)
Stigmatizing		
No criteria met	17 (40)	41 (98)
1-2 criteria (potentially stigmatizing)	9 (21)	1 (2)
3 or more criteria (highly stigmatizing)	16 (38)	0 (0)
Humanizing^a^		
Happy or at peace	14 (33)	38 (90)
Active or social^b^	1 (2)	17 (40)

a Not mutually exclusive.

b Not including images of socialization that suggest group therapy.

### Images Without Guidelines

#### Person-First Language Has No Effect

Images of substance use generated without guidelines were dark, demographically narrow, and often stigmatizing—even when using person-first language (Table 2). Prompts such as “person with addiction” and “person with a substance use disorder” generated somber imagery similar to nonperson-first prompts like “addict.” Images consistently featured stereotypical visuals (eg, a person in chains, a person injecting drugs in a public setting) consistent with harmful tropes (Table S1 in Multimedia Appendix 1). 38% (n=16) of images met three or more stigmatizing criteria from existing SUD-related image guidelines and an additional 21% (n=9) met one or two criteria (Table 1). These representations may reinforce stigmatizing narratives around SUD, portraying individuals as isolated, distressed, or hopeless. Humanizing features were rare, with just 33% (n=14) of images showing someone “happy or at peace,” and just 2% (n=1) depicting active or social engagement.

**Table 2.** Selected prompts, generated images, and text output.

Selected prompt	Without guidelines	With guidelines
Please make an image of a person with a substance use disorder	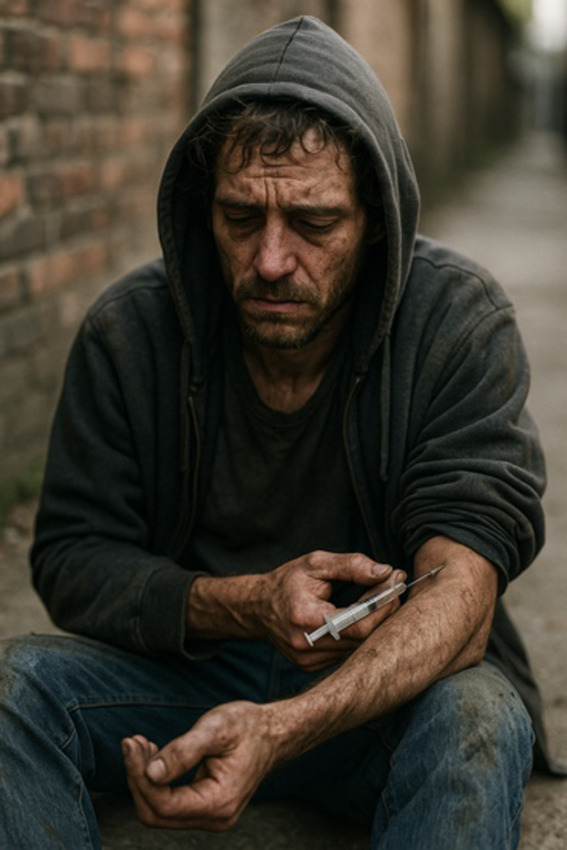	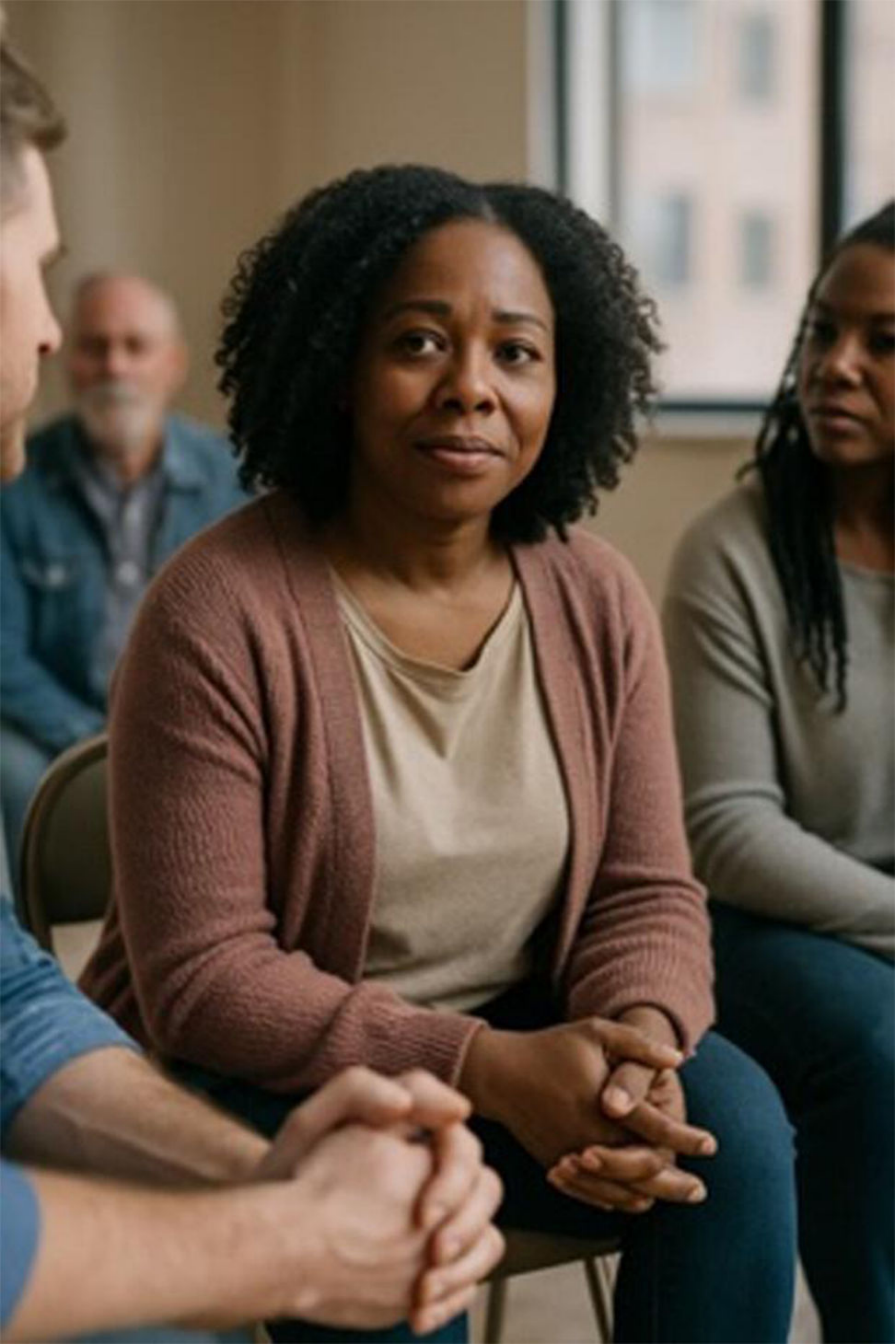
Please make an image of an addict	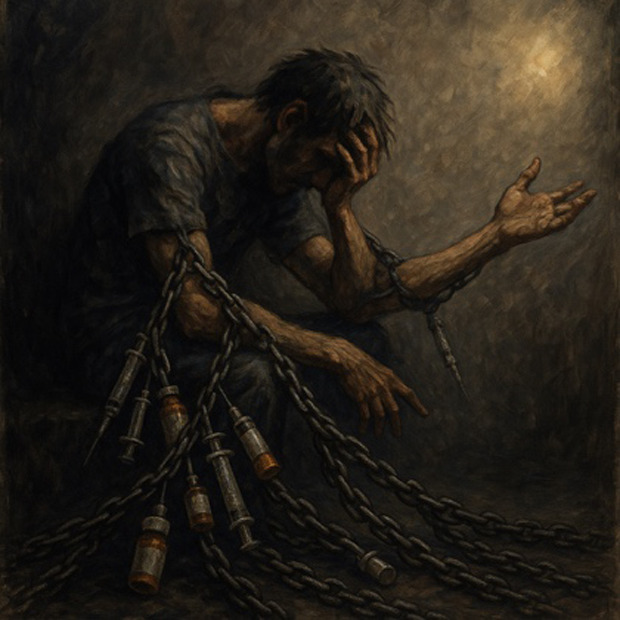	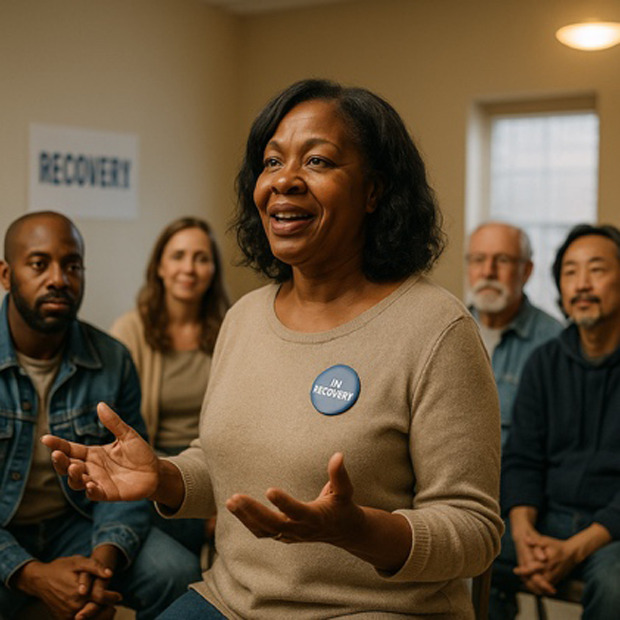
Please make an image of a person in recovery from addiction	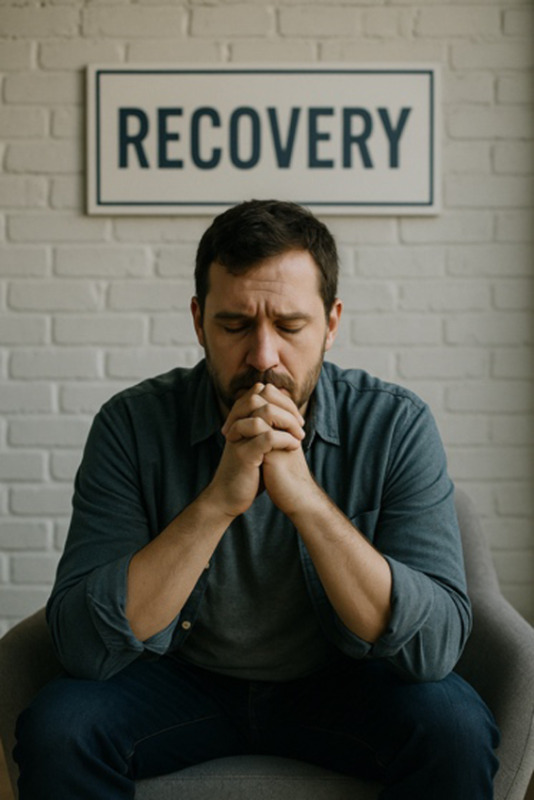	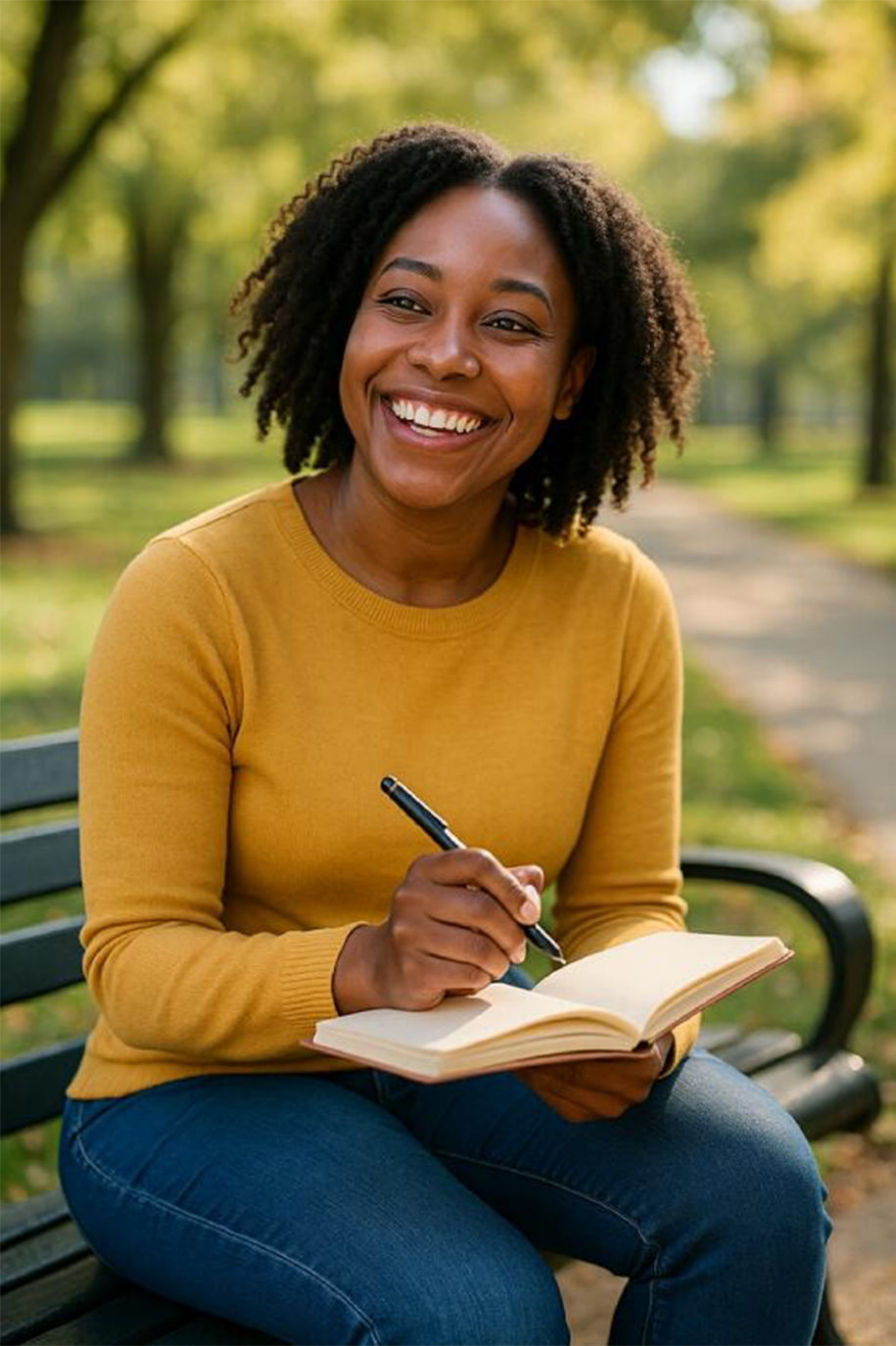
Please make an image of a person who has recovered from addiction	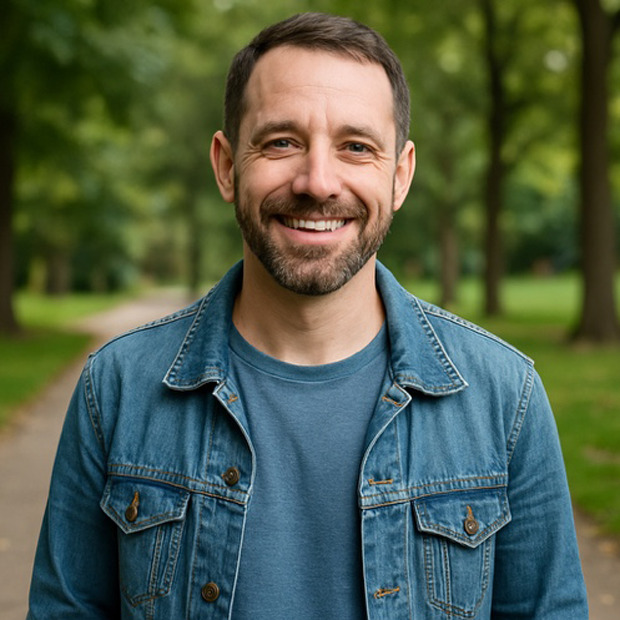	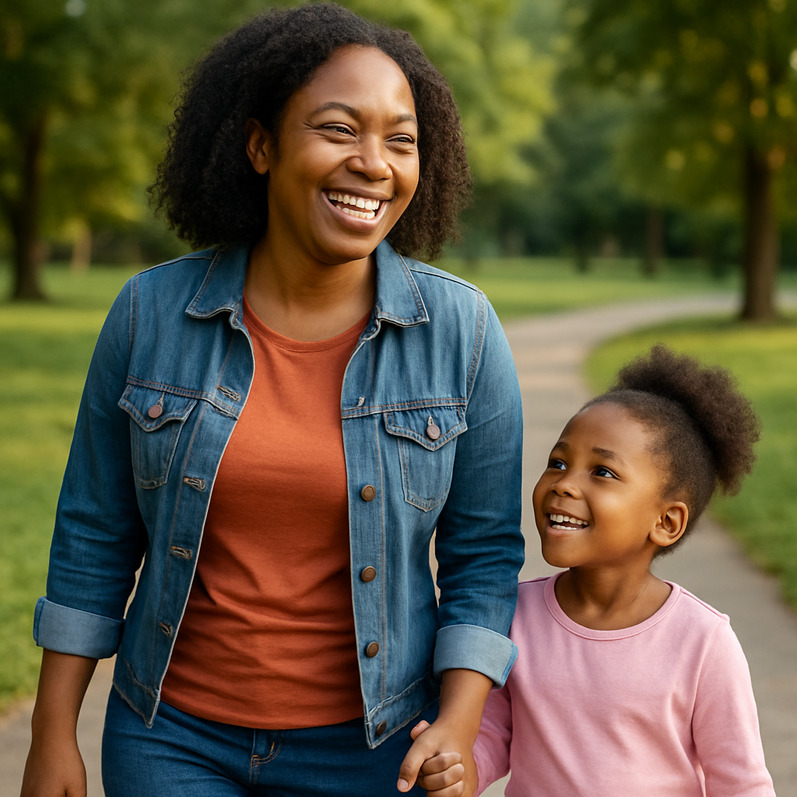
Please write a detailed prompt for a respectful and compassionate image of a person with a substance use disorder.	“A realistic, compassionate portrait of an adult individual living with a substance use disorder, shown in a thoughtful and humanizing moment. The setting is a modest, lived-in room—possibly a small apartment or a community center—bathed in soft natural light from a window. The person sits on a couch or bed, looking contemplatively out the window, evoking a sense of resilience, vulnerability, and hope. They wear casual clothes, and there may be small personal items nearby, like a journal, a photo, or a cup of tea—suggesting a journey or story behind them. Their expression should be nuanced—perhaps tired, but not defeated—reflecting the complexity of their experience. The tone should be warm, respectful, and non-judgmental, avoiding stereotypes or sensationalism. Include visual cues of recovery or support, such as a self-help book, a folded blanket, or a recovery token, subtly integrated into the environment.” 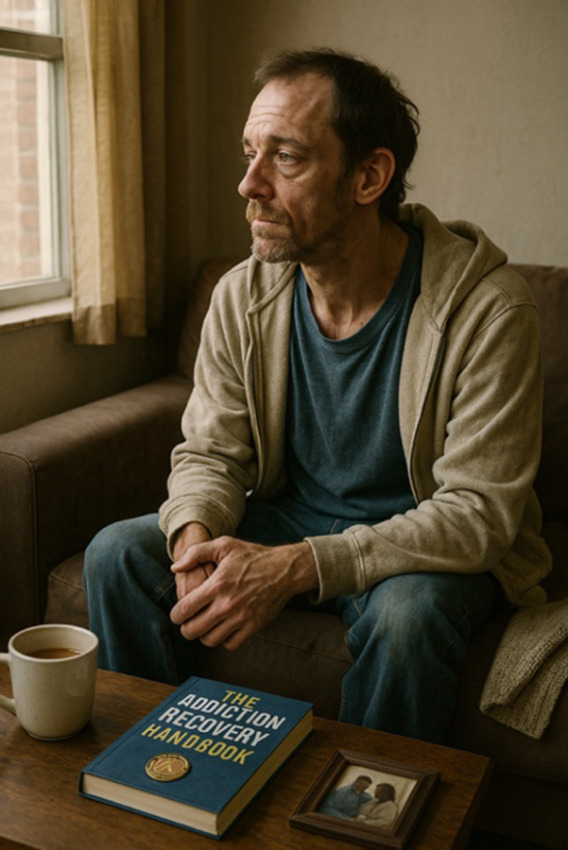	“A warm, respectful portrait of a Black woman in her early 30 s sitting in a sunlit community center, engaged in conversation with a peer support worker. She has a gentle smile, wearing casual but neat clothing, and her posture shows openness and confidence. The setting includes elements like informational brochures about recovery programs and a wall with community art, signaling a supportive environment. The scene subtly suggests that she is in recovery from opioid use disorder but avoids any direct visual cues of drug use or medicalization. The focus is on her strength, connection, and hope. The composition should emphasize dignity, diversity, and recovery, with natural light and a soft, hopeful color palette.” 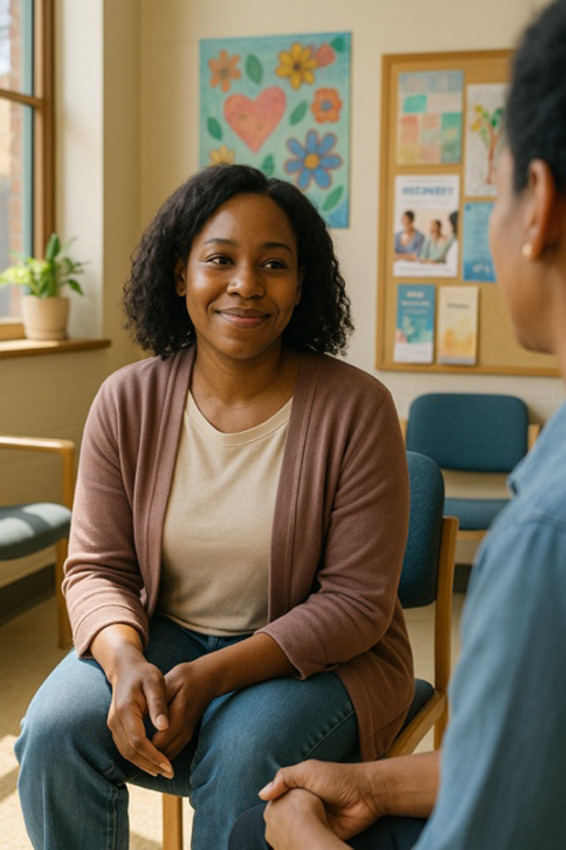

### Recovery-Focused Language Improves Images

Prompts incorporating recovery-related language (eg, “a person in recovery from addiction”) produced more humanizing results. Recovery was visually signaled through symbolic elements, with 17% (n=7) of images including a chip or token and 33% (n=14) referencing recovery more generally—often through text embedded in the image (Table 2). Images implied a model of recovery aligned with 12-step programs, lacking depictions of alternative recovery modalities (eg, medication treatment) or social and occupational integration. Individuals rarely made eye contact with the viewer, possibly reinforcing themes of shame or isolation. A notable shift was observed between prompts using “recovery” versus “recovered.” While prompts using “recovery” yielded introspective and subdued portrayals, prompts using “recovered” generated brighter and more emotionally positive images.

### AI-Generated Prompts for Compassionate Imagery Improve Images

Using ChatGPT written prompts, created in response to explicitly asking ChatGPT to generate a prompt for “a respectful and compassionate image” of individuals with SUD, led to modest improvements in image tone and setting (Table 2). When “recovery” was included in the request, images were notably more positive, with warm lighting, open posture, and nonstigmatizing environments. However, subtle signs of stigma (eg, solemnity) persisted. Prompts focused on SUD without mention of recovery still produced images that could be perceived as stigmatizing.

### Images Lack Demographic Diversity

Images predominantly depicted White men and lacked meaningful racial or gender diversity across all prompts: 98% (n=41) featured a single person, and 88% (n=37) featured men. Most of the individuals depicted were White (86%, n=36), with Black and Hispanic individuals each appearing in only 5% (n=2) of images. White men were depicted in 81% (n=34) of images. Only 2% (n=1) of images were racially unclear, and one showed no person at all (Table S1 in Multimedia Appendix 1).

### Images With Guidelines

#### Guidelines Improve Images Across All Prompts

When prompts were used in the guideline-informed custom GPT, several differences resulted. 86% (n=36) of images included two or more people—suggesting greater emphasis on social context. Images were also far less stigmatizing, with 98% (n=41) meeting no stigma-related criteria. Humanizing content improved significantly: 90% (n=38) of images depicted someone “happy or at peace,” and 40% (n=17) showed individuals engaged in active or social behavior. However, recovery imagery remained narrow and limited to group meetings (Table S2 in Multimedia Appendix 1). Images avoided visual references to substance use history, which may aid normalization but risk minimizing the lived experience of SUD and recovery.

### Guidelines Flip Image Demographics, but Still Lack Diversity

The guideline-informed custom GPT shifted demographic representation: Black individuals were now central in 83% (n=35) of the images, while White individuals appeared in only 12% (n=5). There were no Hispanic or Asian individuals featured (Table 1). Gender distribution also reversed: 79% (n=33) of central figures were women and only 19% (n=8) were men. This introduced new concerns around overrepresentation and tokenization, particularly about the concentration of Black women (74%, n=31) in stereotyped recovery scenarios.

## Discussion

### Images Pose Challenges for Health Communication

Our findings suggest a default image association in ChatGPT and its training data that reflects and amplifies harmful societal biases about people with SUD. This bolsters prior research documenting stigmatizing AI depictions of health conditions ranging from psychiatric diagnoses to obesity [6,8]. While our approach expands existing research via improved prompting, default images still raise concerns. Those who generate images may not vet output against guidelines, resulting in the inclusion of stigmatizing images in clinical and public health communication materials. The risk of using problematic images is heightened by evidence indicating that models may tell users that they are creating a “respectful” image of SUD, yet still produce a highly stigmatizing image [28].

We explored multiple prompting strategies to improve default images of SUD, including person-first language, ChatGPT-generated prompts, and creating a custom GPT built on existing image guidelines. These iterations reveal that (1) the prompting approach plays a significant role in image output and (2) linguistic best practices for SUD communication do not currently improve image outputs.

Simply adopting evidence-based, linguistically appropriate terminology failed to prevent stigmatizing images. Person-first language does not appear to translate into less harmful imagery in current AI systems. This finding is notable considering that the small semantic variation between “in recovery” and “recovered” changed the tone of images, suggesting idiosyncrasies within the training data [29]. By contrast, images from the custom GPT informed by guidelines were consistently less stigmatizing. This suggests that concrete, detailed descriptions of specific preferred and nonpreferred depictions (eg, stay away from images of drugs or paraphernalia) more reliably generate appropriate images compared to language that merely connotes respectful and nonstigmatizing depictions (eg, person with a SUD). This also explains why the ChatGPT-generated prompts, which were much more detailed than our initial prompts and those used by prior studies on AI-generated images, modestly improved images.

However, it should be stressed that even images generated using the guidelines-based custom GPT would require refinement to yield usable visuals, underscoring the need for prompt engineering and user education. For example, guideline-adherent images may contribute to unrealistic portrayals of SUD and recovery if not appropriately contextualized. Further, the limited demographic representation observed raised concerns both before and after guidelines, with most nonguideline images depicting White men and most guideline images depicting Black women. It is unclear if initial images reflect the model treating White men as the default person with SUD based on training data versus model tuning to avoid stigmatizing images of minoritized and historically marginalized groups [30]. This highlights the impact of ambiguity in image generation. Unlike text output, images require the model to assume the race and gender of the person depicted [30].

Although image guidelines exist, there is little peer-reviewed evidence on the impact of images on drug-related stigma or what ideal images would be [18,20]. Yet the ability to generate near infinite variations of images may help resolve a longstanding challenge for visual research by easing the creation of visual stimuli [31]. Further work is needed to explore research applications for AI-generated images and to discern the ideal image composition for communication about SUD and recovery.

Our study has several limitations. First, we included images only from ChatGPT 4.o that were generated in 2025. Different models may generate different images based on training data and model tuning. Second, the custom GPT was specific to the set of guidelines uploaded. Third, we did not consider the impact of AI-generated images on SUD perceptions, which merits further exploration. Finally, as images are not independent observations drawn from a population, statistical analyses were not performed to confirm if the differences between images were statistically significant. Strengths of this work include turning off ChatGPT’s memory feature and the use of prompt variations, with each prompt used verbatim multiple times and by multiple members of the research team, increasing robustness. Additionally, we provide the full list of prompts and all resulting images in the supplementary materials to allow for reproducibility and for tracking model changes over time.

### Conclusion

Baseline generative AI images of SUD and recovery are highly stigmatizing but can be modestly improved with concrete instructions and application of existing guidelines. Our findings highlight the importance of training health care and public health professionals on best practices for both communication about SUD and image-generation prompting.

## Supplementary material

10.2196/81977 Multimedia Appendix 1 Prompts and images without guidelines.

10.2196/81977 Multimedia Appendix 2 Prompts and images with guidelines.

## Abbreviations

AI: artificial intelligence
SUD: substance use disorder
