# Supplementary material for: AI-Generated Images of Substance Use and Recovery: Mixed Methods Case Study
Source: JMIR AI. 2026 Feb 19;5:e81977. doi: 10.2196/81977 (PMC12919905; doi:10.2196/81977)
Supplement: Multimedia Appendix 1 [file ai-v5-e81977-s001.docx]

**Appendix Table S1.** Prompts and Images Without Guidelines

| **Prompt** | **Researcher 1** | **Researcher 2** | **Researcher 3** |
| --- | --- | --- | --- |
| Please make an image of substance use disorder | 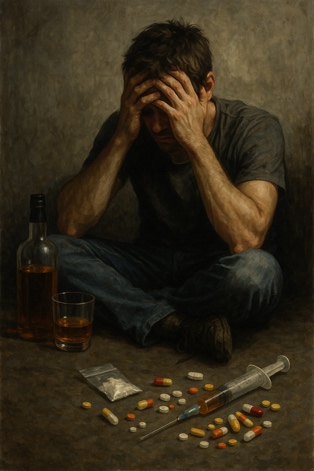 | 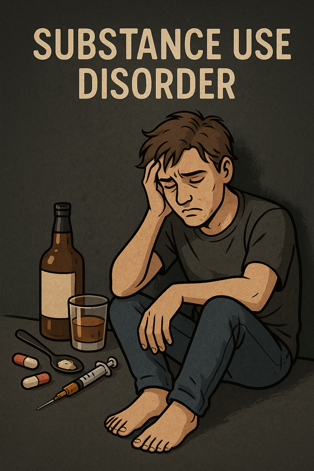 | 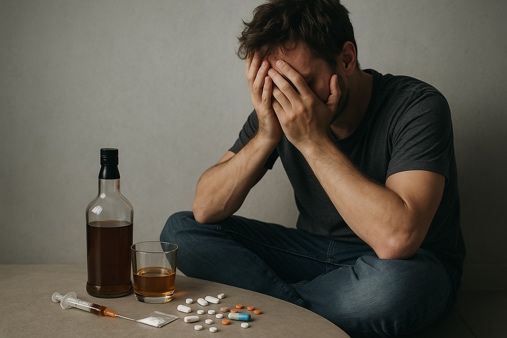 |
| Please make an image of addiction | 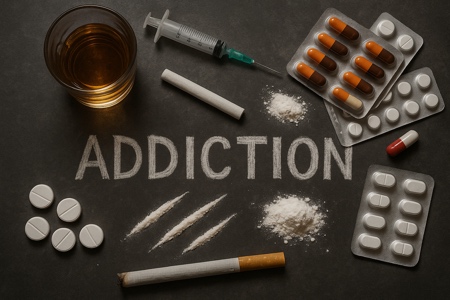 | 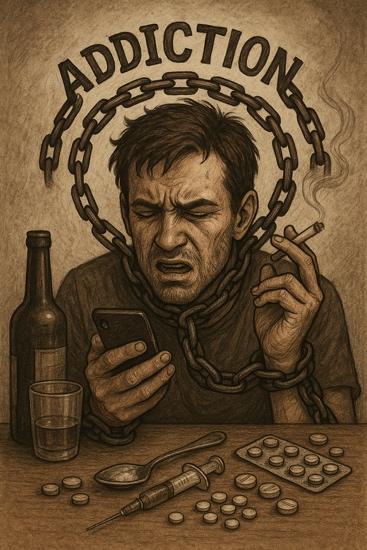 | 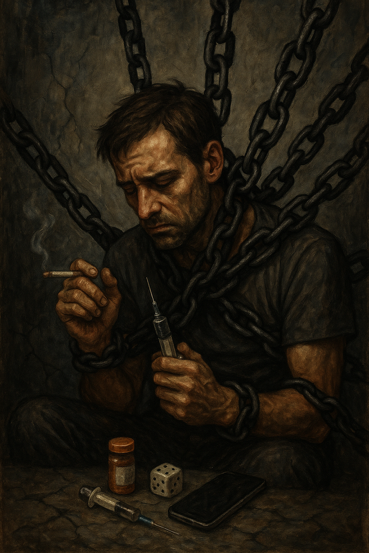 |
| Please make an image of being in recovery | 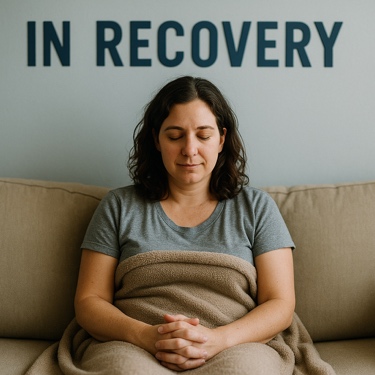 | 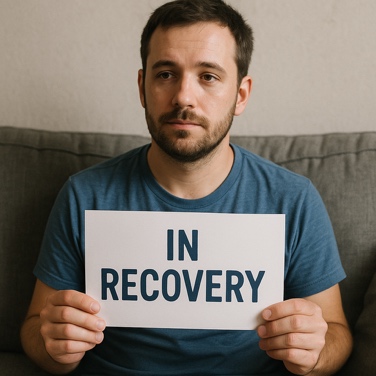 | 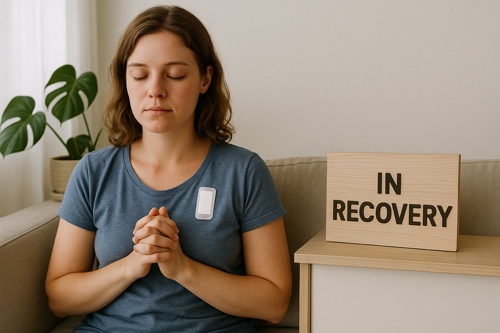 |
| Please make an image of a p**erson with a substance use disorder** | 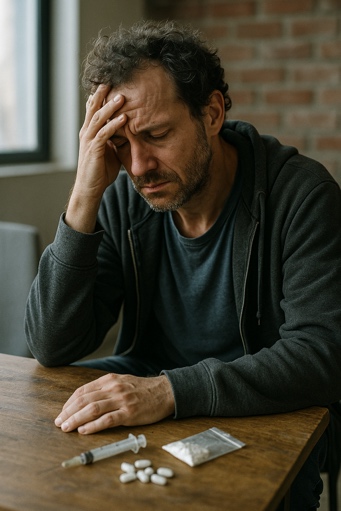 | 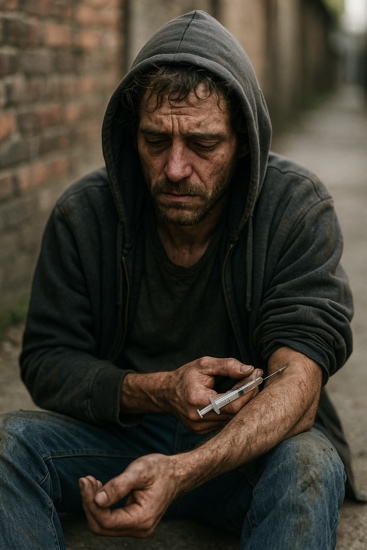 | 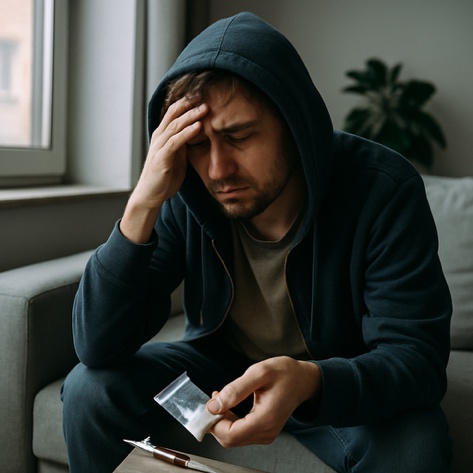 |
| Please make an image of a p**erson with addiction** | 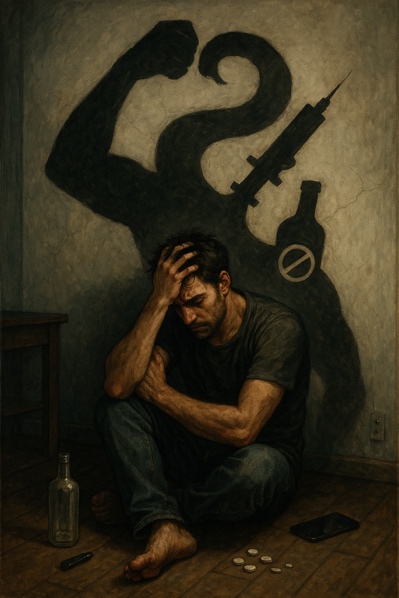 | 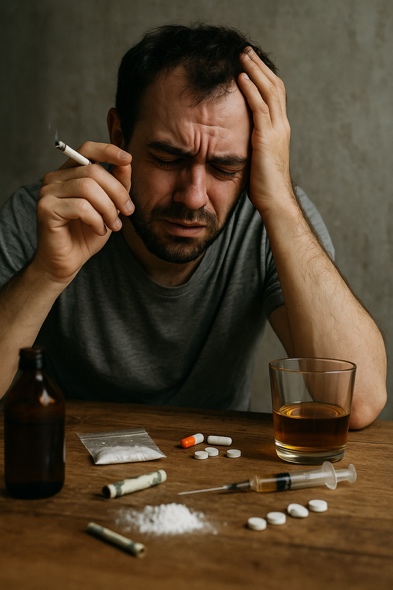 | 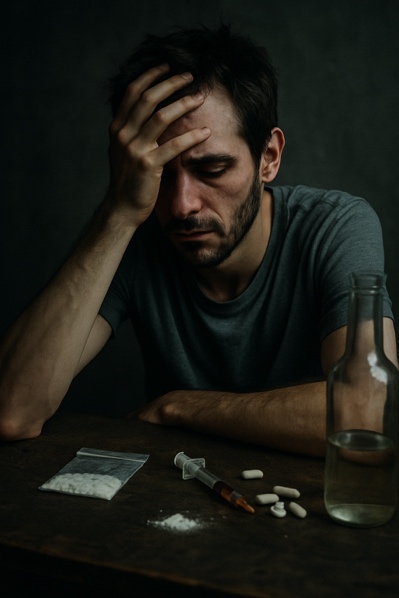 |
| Please make an image of an addict | 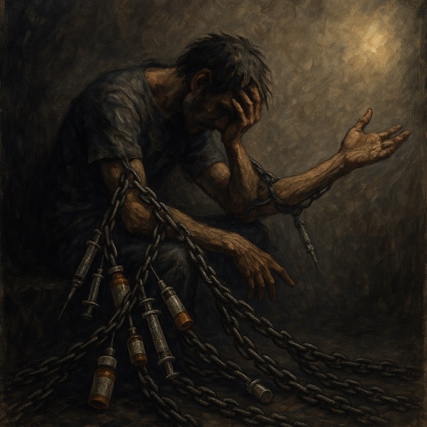 | 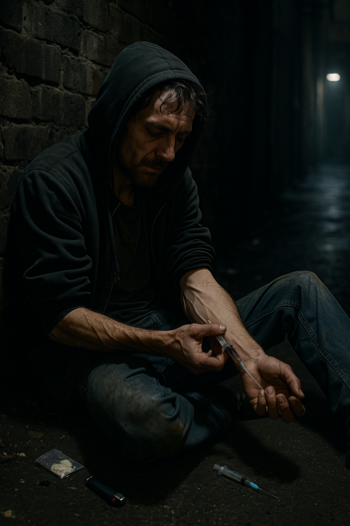 | 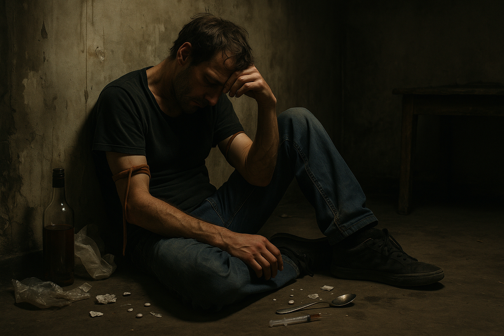 |
| Please make an image of a p**erson in recovery from a substance use disorder** | 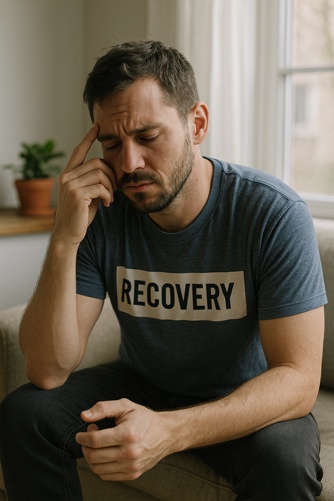 | 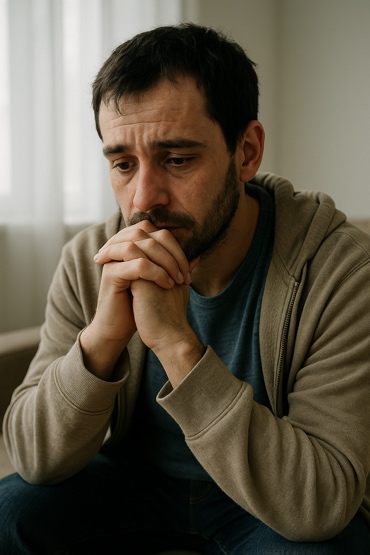 | 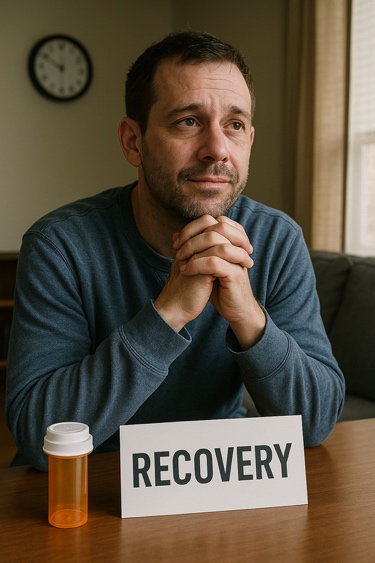 |
| Please make an image of a **person in recovery from addiction** | 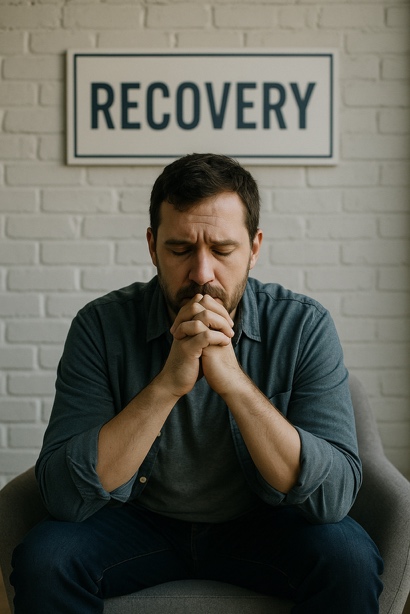 | 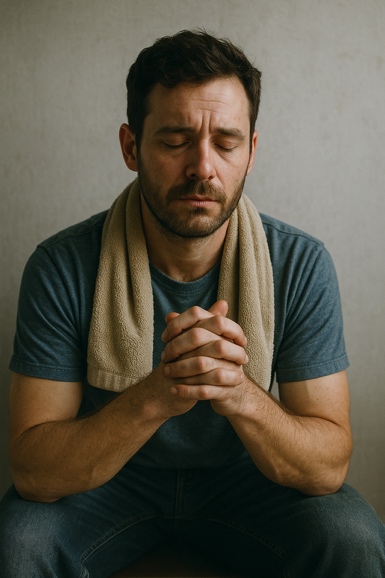 | 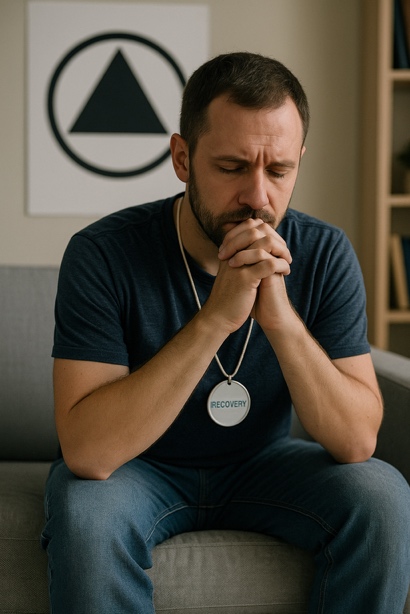 |
| Please make an image of a r**ecovering addict** | 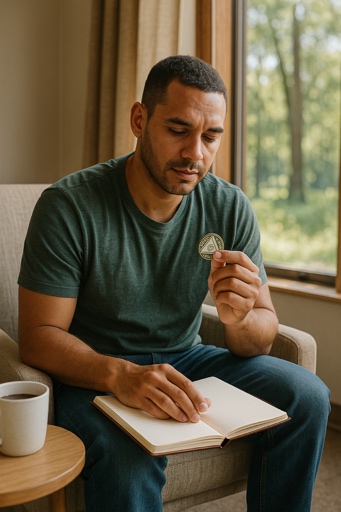 | 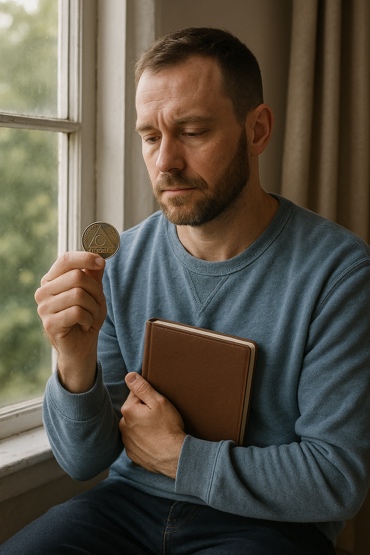 | 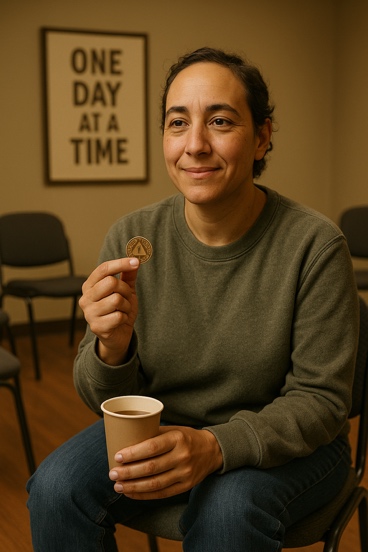 |
| Please make an image of a p**erson who has recovered from a substance use disorder** | 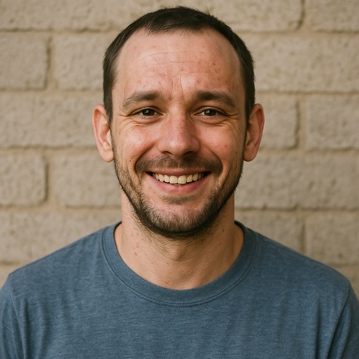 | 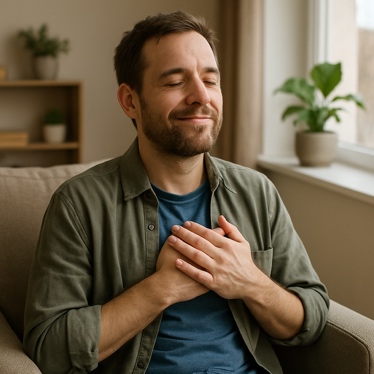 | 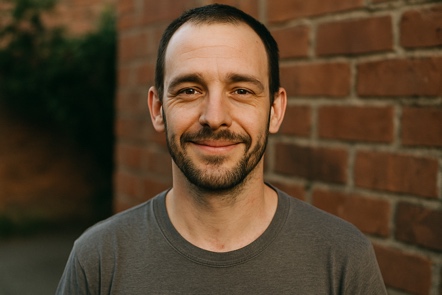 |
| Please make an image of a p**erson who has recovered from addiction** | 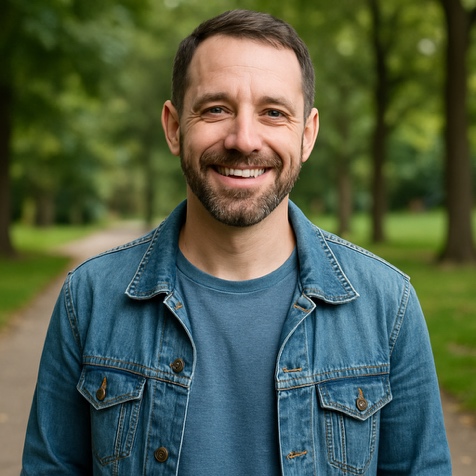 | 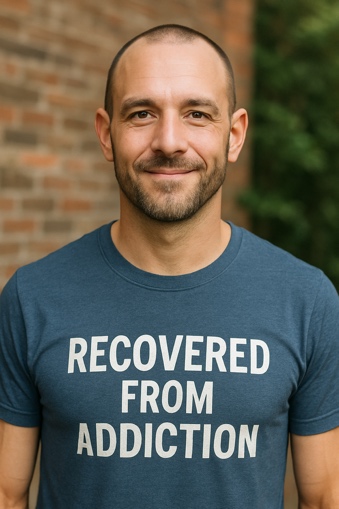 | 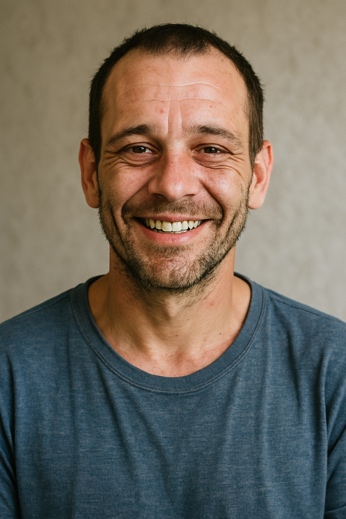 |
| Please make an image of a f**ormer addict** | 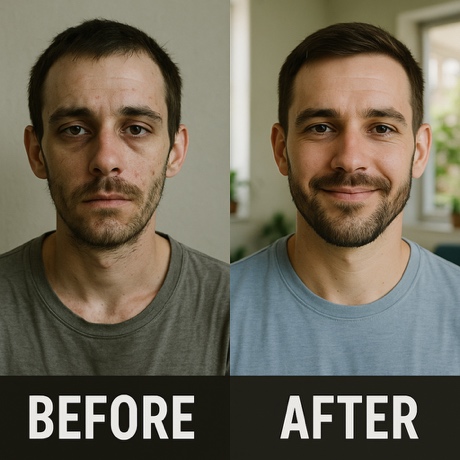 | 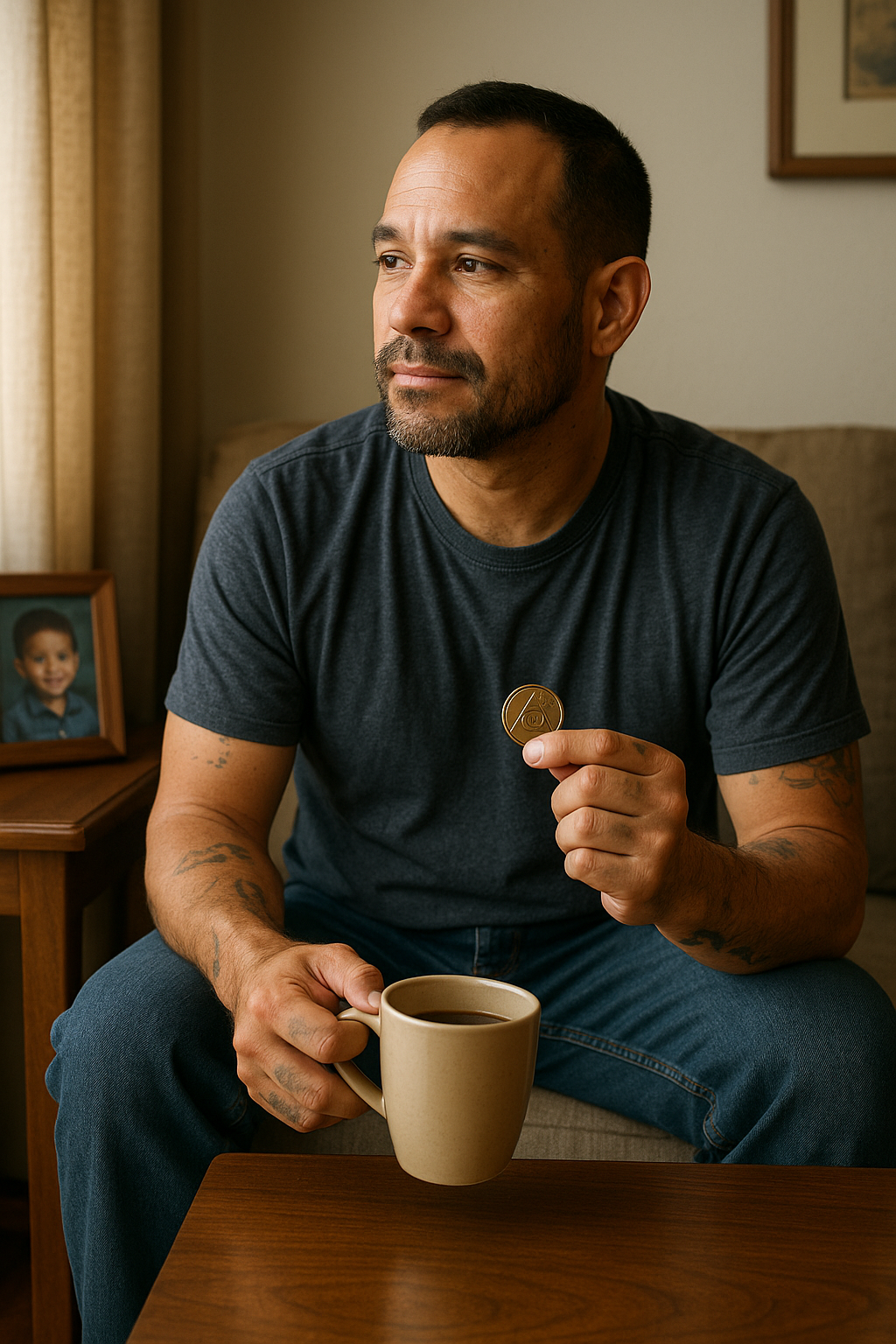 | 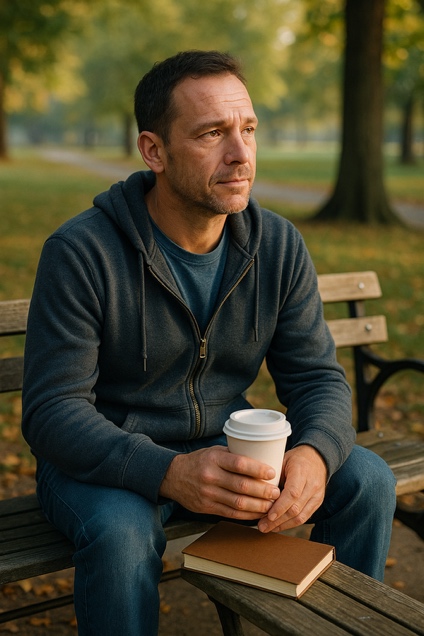 |
| Please write a detailed prompt for a respectful and compassionate image of a person in recovery from a substance use disorder | “A serene and hopeful portrait of a person in recovery from substance use disorder. The person, appearing in their 30s or 40s, has a gentle and calm expression, showing strength, dignity, and quiet resilience. They are seated on a park bench or inside a peaceful, sunlit room, dressed in clean, casual clothes. Subtle visual cues—like a recovery journal in hand, a recovery coin or bracelet, or a cup of tea—suggest their journey without being explicit or stereotypical. The environment is warm and uplifting, with natural light streaming in, soft shadows, and elements of nature or comfort nearby (like a tree, flowers, or an open window). The focus is on inner peace and growth, not struggle. The overall tone is respectful, affirming, and compassionate, emphasizing the humanity, hope, and strength of the individual.” | “Create a respectful, compassionate, and hopeful image of a person in recovery from a substance use disorder. The person should be portrayed with dignity and strength. Depict them sitting peacefully in a cozy, sunlit room or a nature setting, such as a park or a quiet forest trail. Their posture is calm and grounded, perhaps with a slight, genuine smile. They may be journaling, meditating, or simply reflecting while holding a warm cup of tea or coffee. Subtle visual elements can suggest their recovery journey—such as a “one day at a time” bracelet, a support book nearby, or framed inspirational quotes in the background. The person should appear healthy and at ease, wearing comfortable clothing. Use warm, natural lighting to enhance the sense of hope and renewal. Avoid any stereotypical or stigmatizing imagery—focus on portraying resilience, humanity, and forward momentum. Ethnicity, age, and gender can be diverse and should reflect authentic representation.” | “A compassionate and respectful portrait of a person in recovery from substance use disorder. The individual is sitting peacefully in a sunlit room, with soft natural light filtering through a nearby window, symbolizing hope and renewal. Their expression is calm, resilient, and reflective—showing strength without struggle. They are casually dressed in comfortable, clean clothing, with subtle symbols of recovery around them, such as a journal, a recovery medallion, or a cup of tea. The background may include small plants, a bookshelf, or a cozy chair—elements that reflect stability and healing. The overall mood is one of dignity, quiet strength, and forward-looking hope. No signs of distress, stigma, or active substance use; instead, focus on emotional recovery, inner peace, and personal growth.” |
|  | 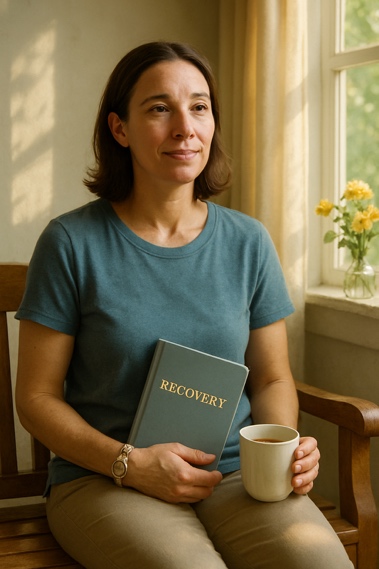 | 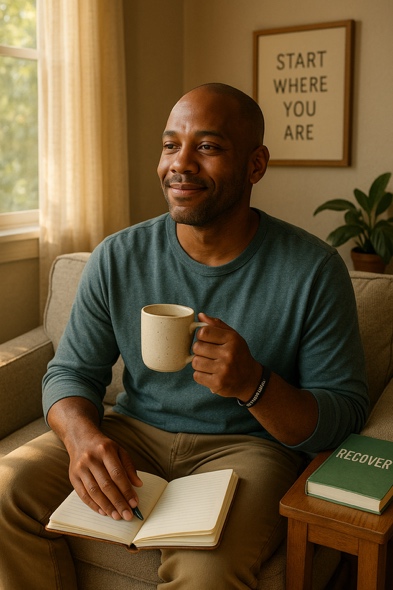 | 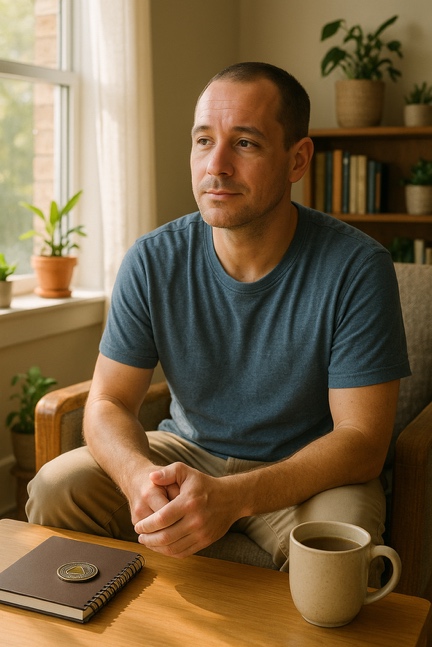 |
| Please write a detailed prompt for a respectful and compassionate image of a person with a substance use disorder | “A realistic, compassionate portrait of an adult individual living with a substance use disorder, shown in a thoughtful and humanizing moment. The setting is a modest, lived-in room—possibly a small apartment or a community center—bathed in soft natural light from a window. The person sits on a couch or bed, looking contemplatively out the window, evoking a sense of resilience, vulnerability, and hope. They wear casual clothes, and there may be small personal items nearby, like a journal, a photo, or a cup of tea—suggesting a journey or story behind them. Their expression should be nuanced—perhaps tired, but not defeated—reflecting the complexity of their experience. The tone should be warm, respectful, and non-judgmental, avoiding stereotypes or sensationalism. Include visual cues of recovery or support, such as a self-help book, a folded blanket, or a recovery token, subtly integrated into the environment.” | “A realistic, empathetic portrait of an adult person living with a substance use disorder. The individual is seated alone on a park bench in soft morning light, dressed in clean but worn clothing. Their expression shows a mixture of resilience, vulnerability, and quiet strength. The environment around them is calm and peaceful, with green trees and gentle sunlight filtering through. Their posture is slightly slouched but not defeated—conveying humanity, dignity, and the weight of lived experience. The focus should be on showing the person as someone deserving of care, hope, and understanding, without stereotypes or sensationalism. The tone of the image is warm and introspective, aiming to promote compassion and reduce stigma.” | “A compassionate, respectful portrait of an adult individual living with a substance use disorder. The person appears thoughtful and resilient, sitting in a modest, softly lit environment that suggests a moment of reflection or quiet strength. They wear casual, slightly worn clothing, and their expression is one of vulnerability and hope—not despair. Subtle details in the background (like a recovery book, a support group flyer, or a mug of tea) suggest efforts toward healing and recovery, without being overly symbolic or dramatic. The style should be realistic and warm, avoiding stereotypes, with a focus on human dignity and complexity. The setting could be a community center, a small apartment, or a clinic waiting room—anywhere that feels real and respectful. Diversity in age, gender, and ethnicity is encouraged.” |
|  | 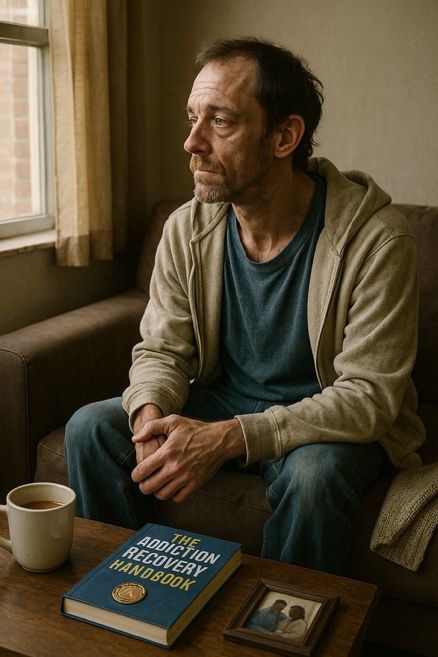 | 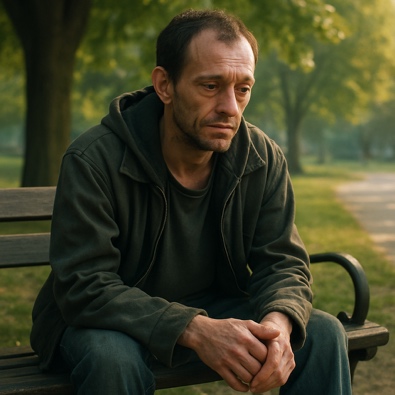 | 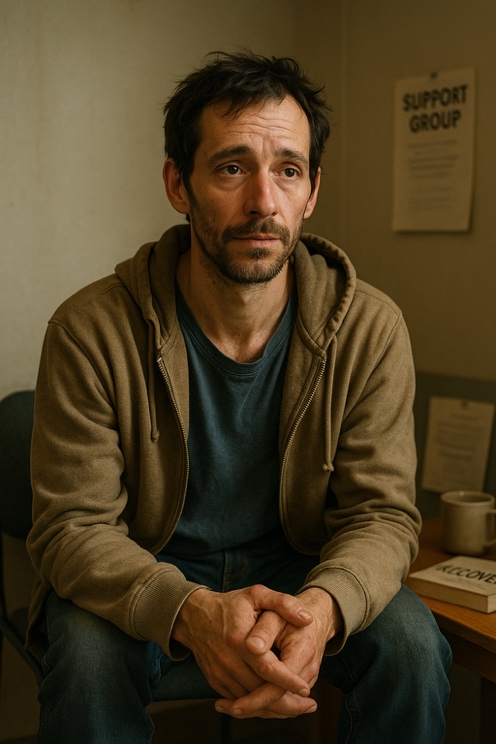 |
