# Supplementary material for: AI-Generated Images of Substance Use and Recovery: Mixed Methods Case Study
Source: JMIR AI. 2026 Feb 19;5:e81977. doi: 10.2196/81977 (PMC12919905; doi:10.2196/81977)
Supplement: Multimedia Appendix 2 [file ai-v5-e81977-s002.docx]

**Appendix Table S2.** Prompts and Images With Guidelines

| **Prompt** | **Researcher 1** | **Researcher 2** | **Researcher 3** |
| --- | --- | --- | --- |
| Please make an image of substance use disorder | 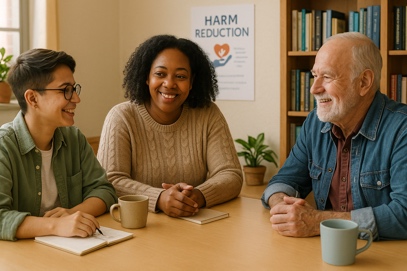 | 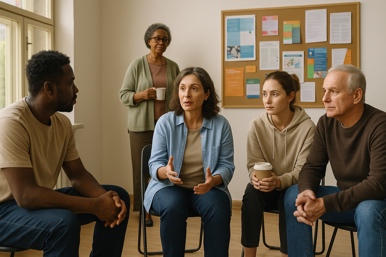 | 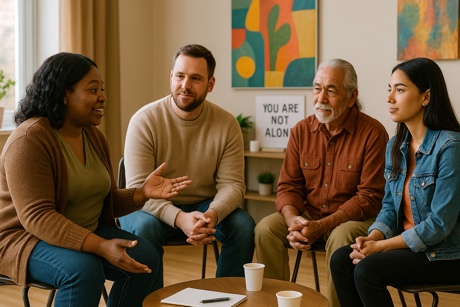 |
| Please make an image of addiction | 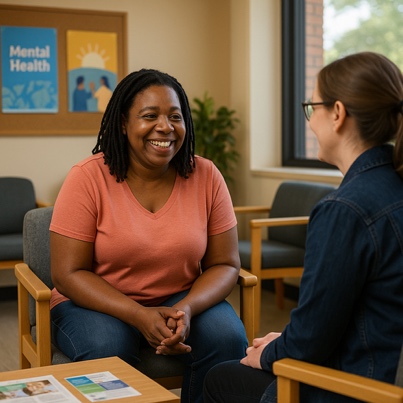 | 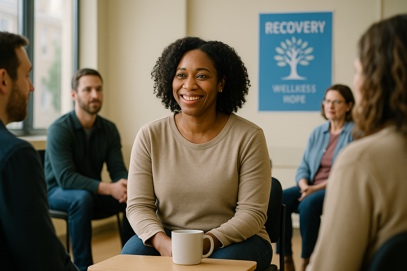 | 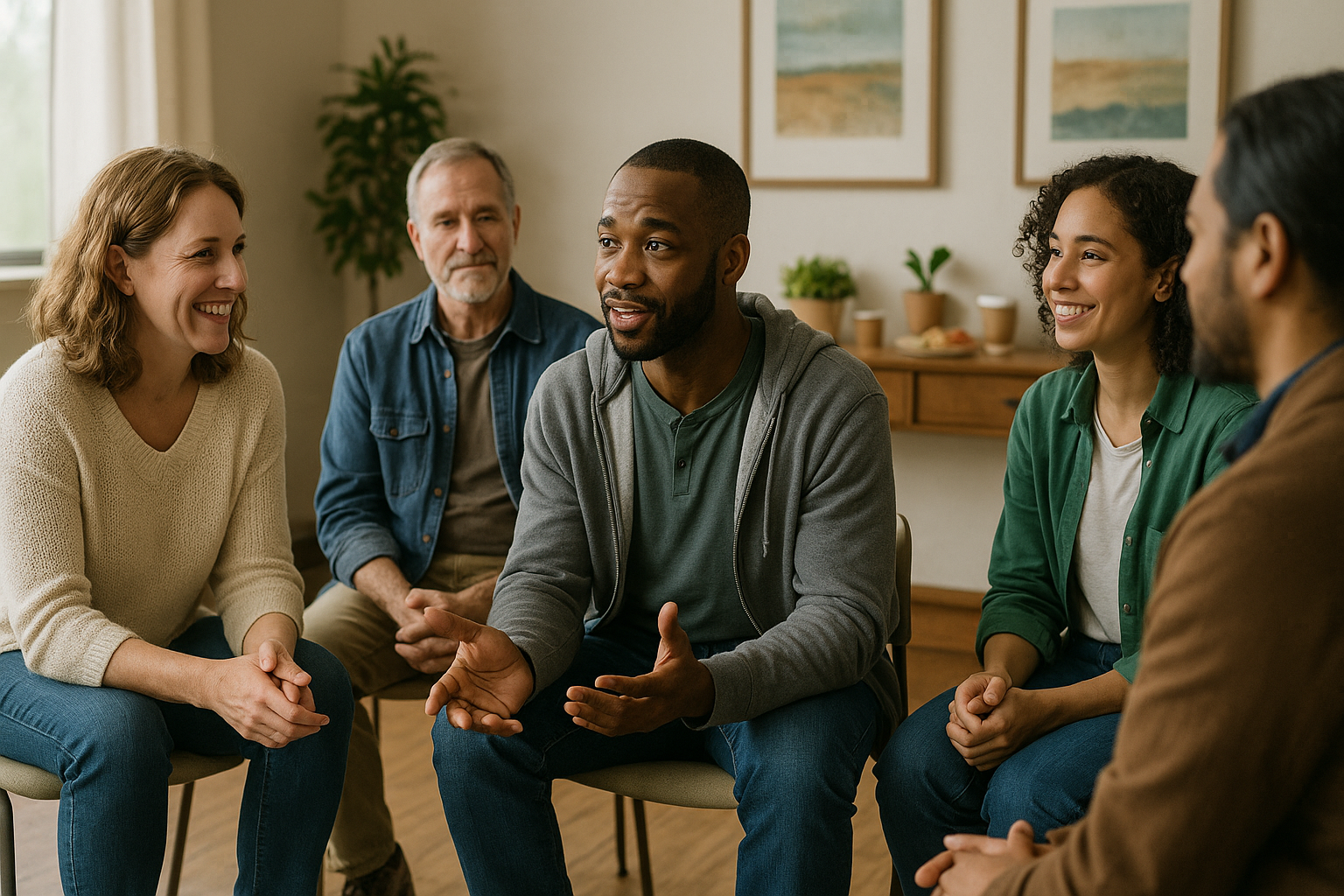 |
| Please make an image of being in recovery | 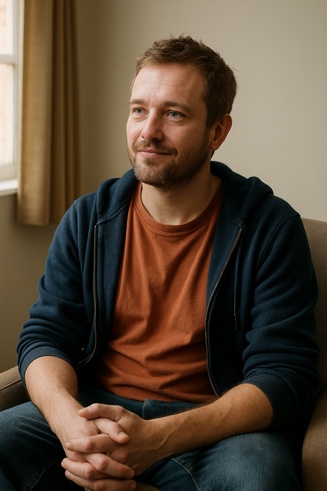 | 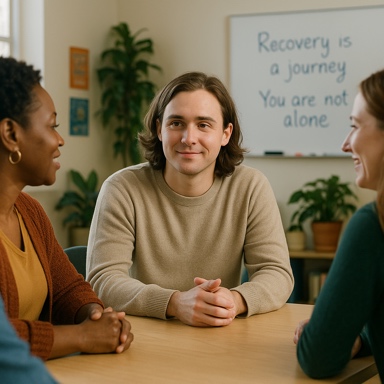 | 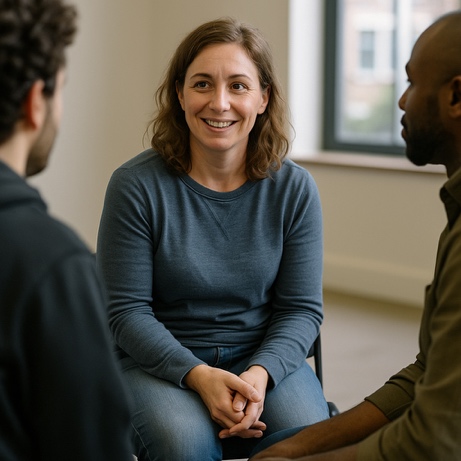 |
| **Please make an image of a person with a substance use disorder** | 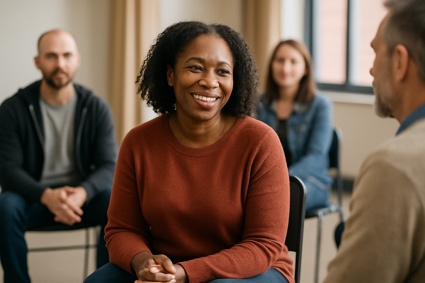 | 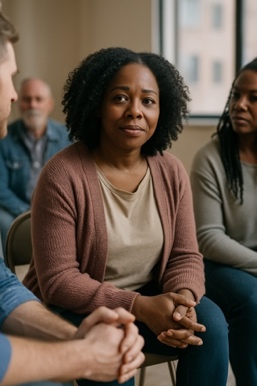 | 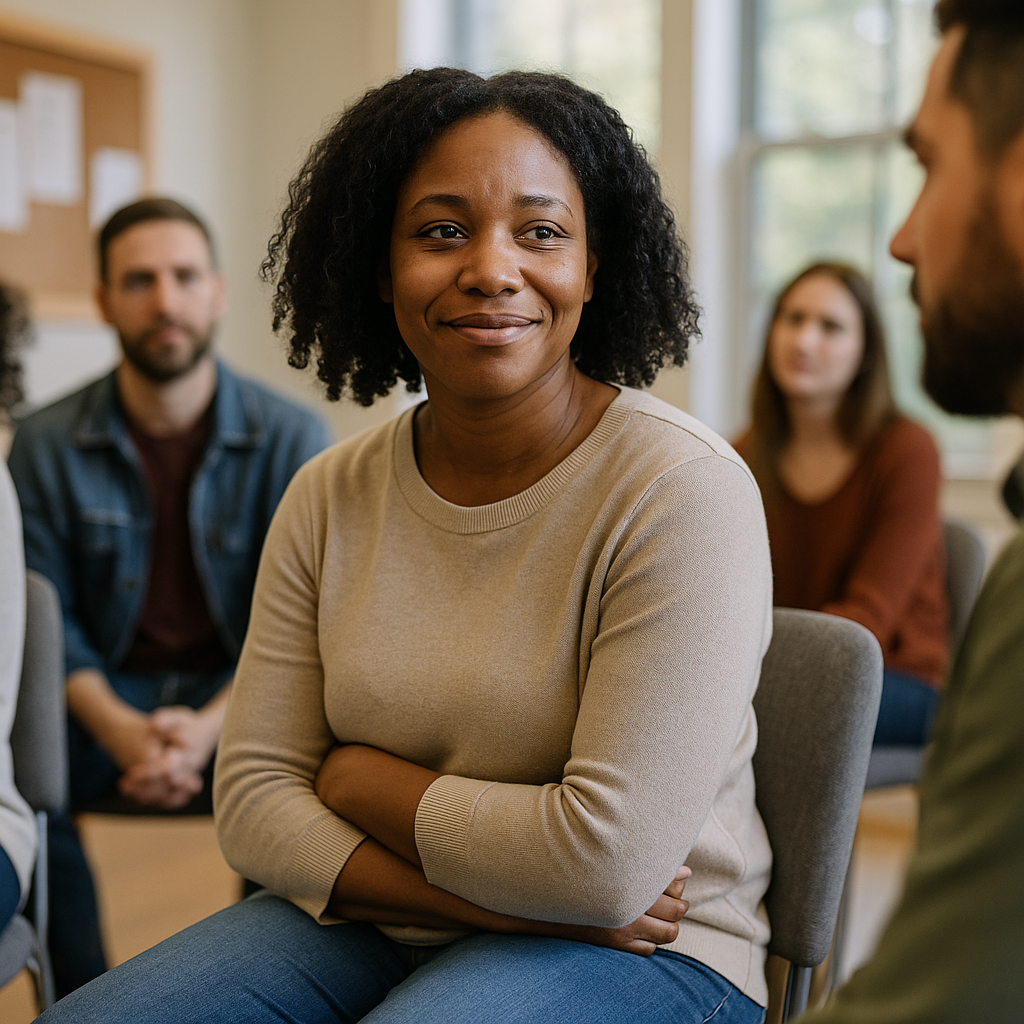 |
| **Please make an image of a person with addiction** | 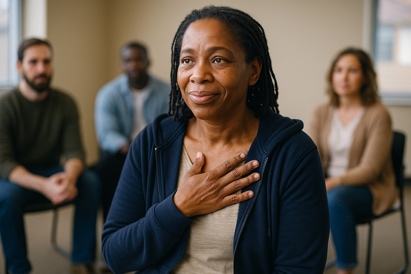 | 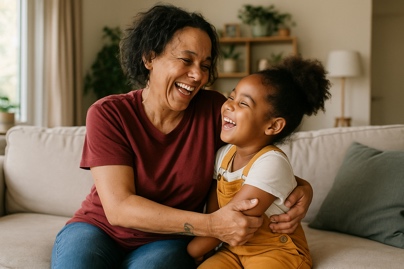 | 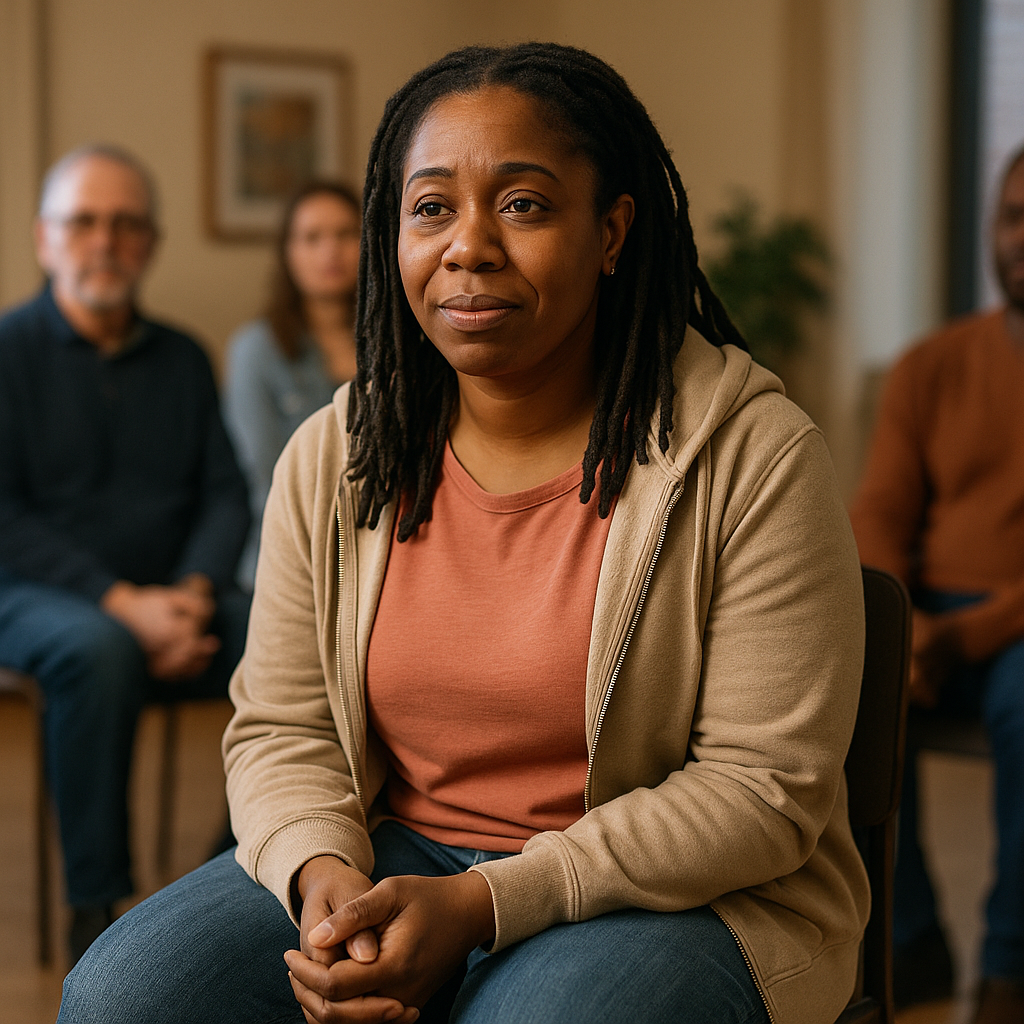 |
| **Please make an image of an** addict | 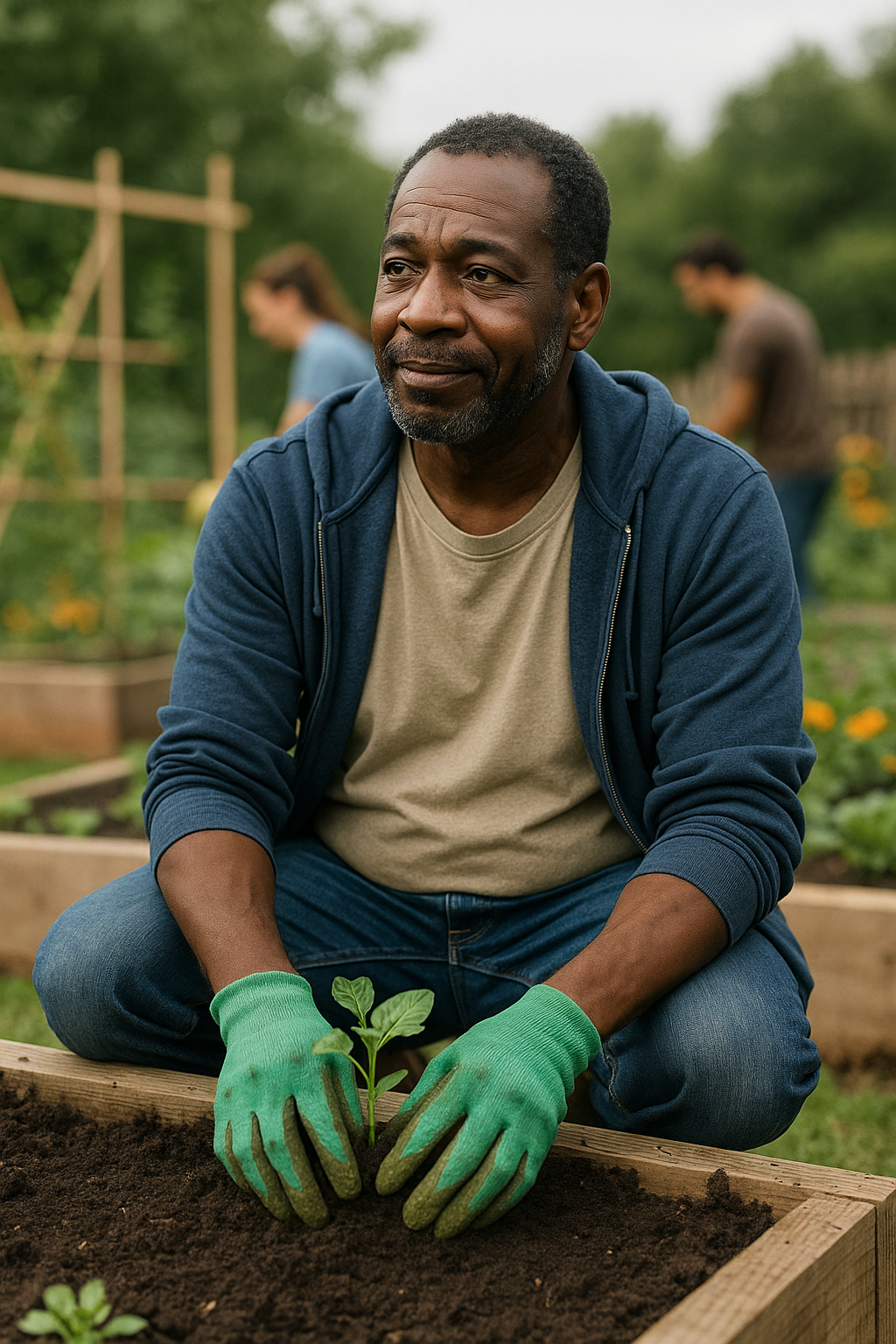 | 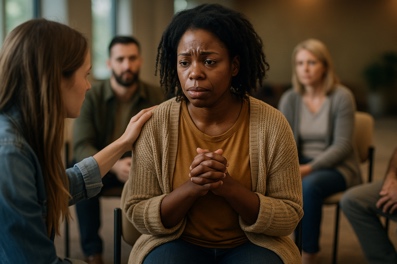 | 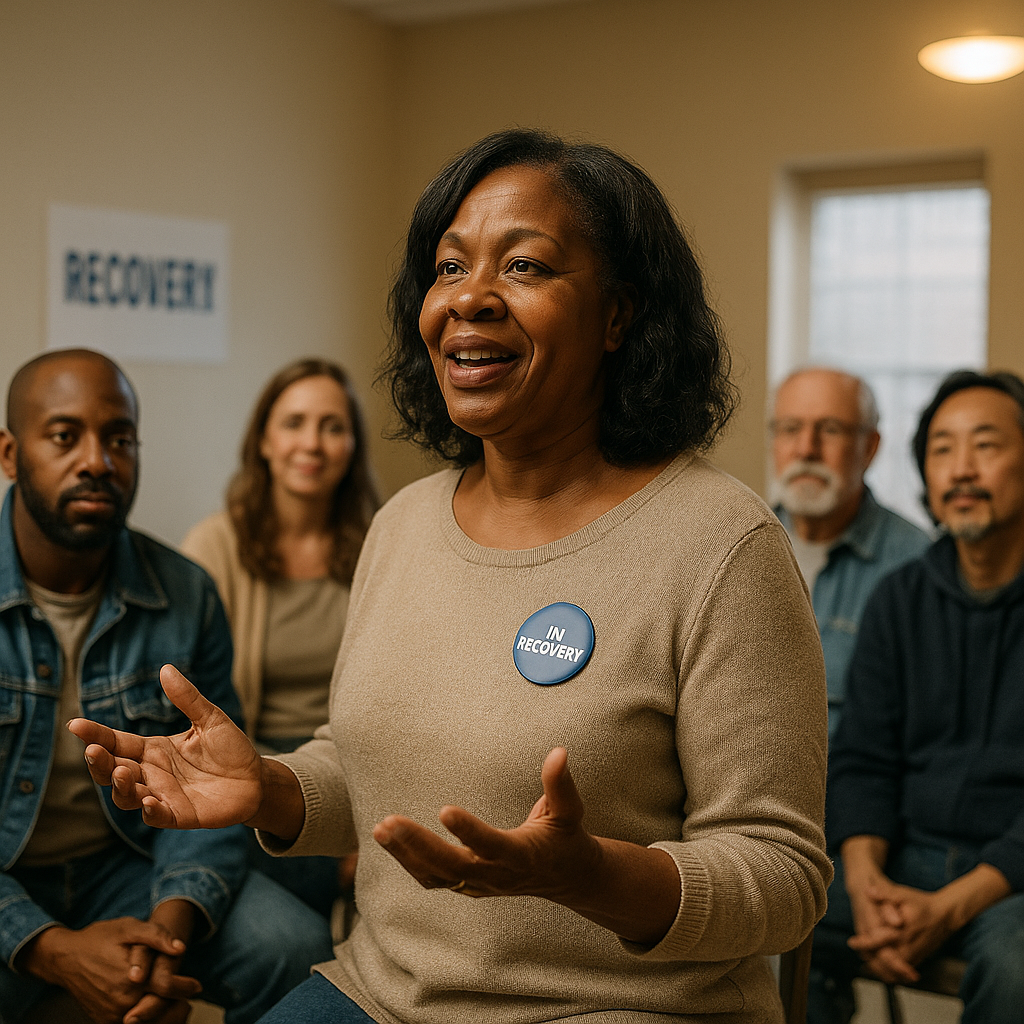 |
| **Please make an image of a person in recovery from a substance use disorder** | 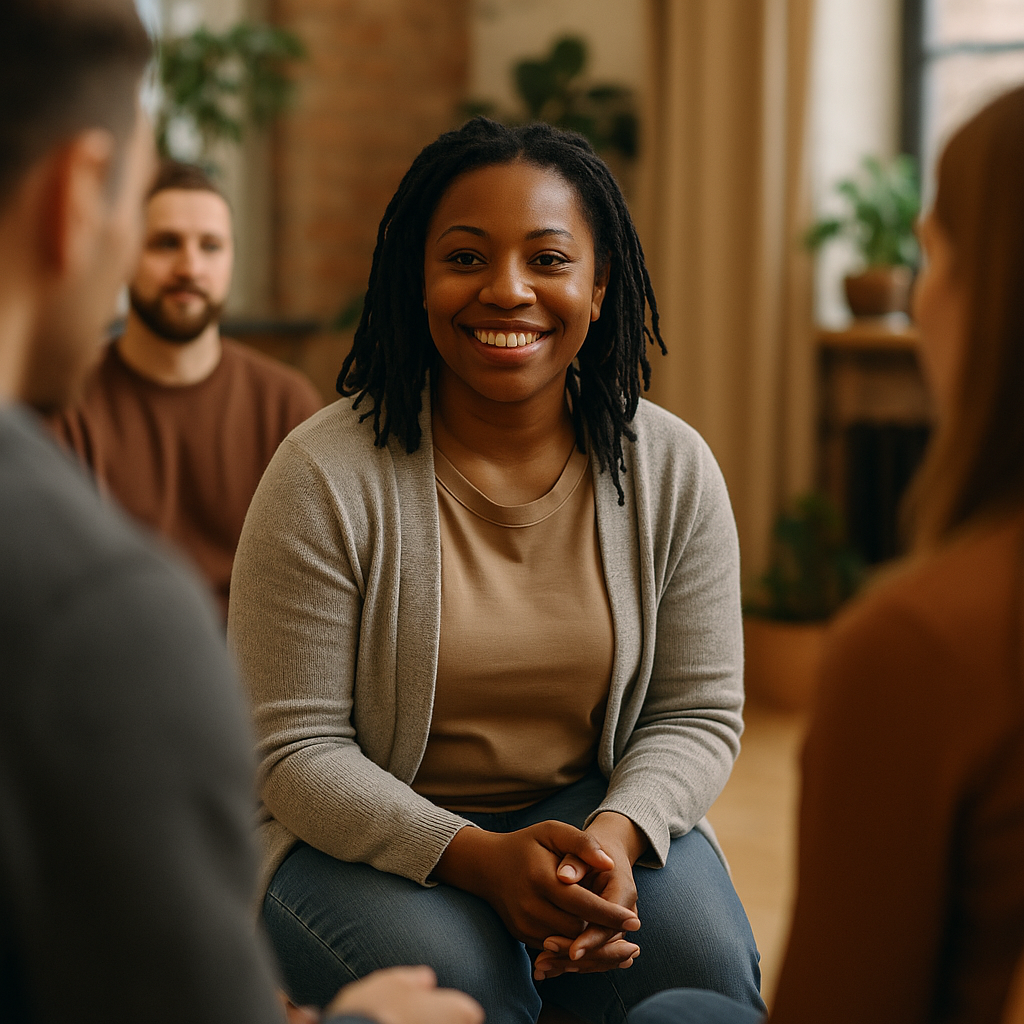 | 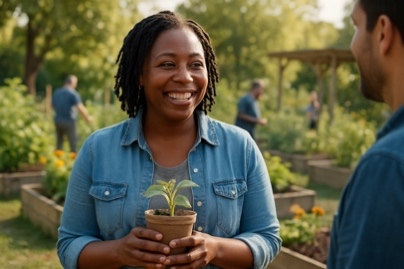 | 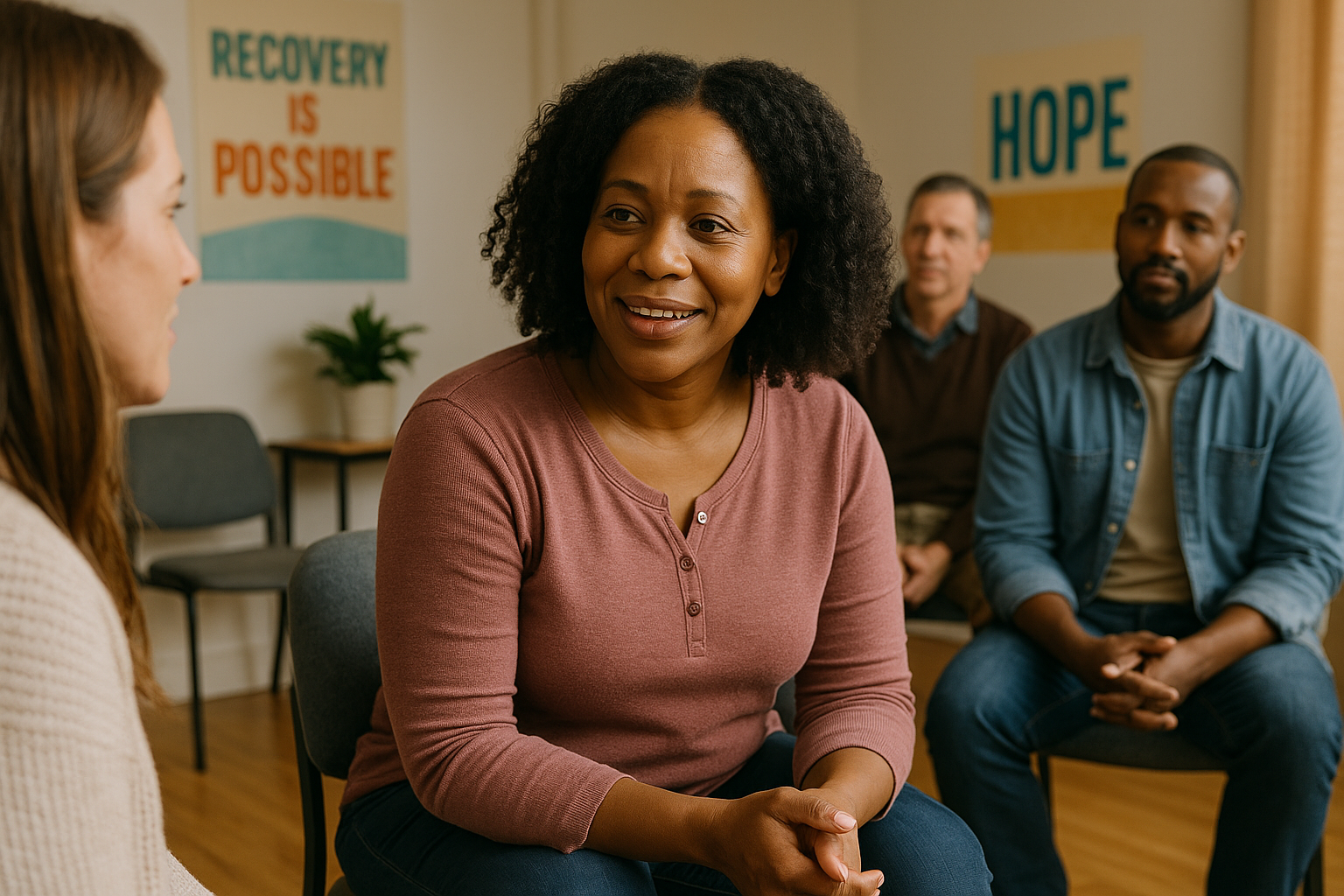 |
| **Please make an image of a person in recovery from addiction** | 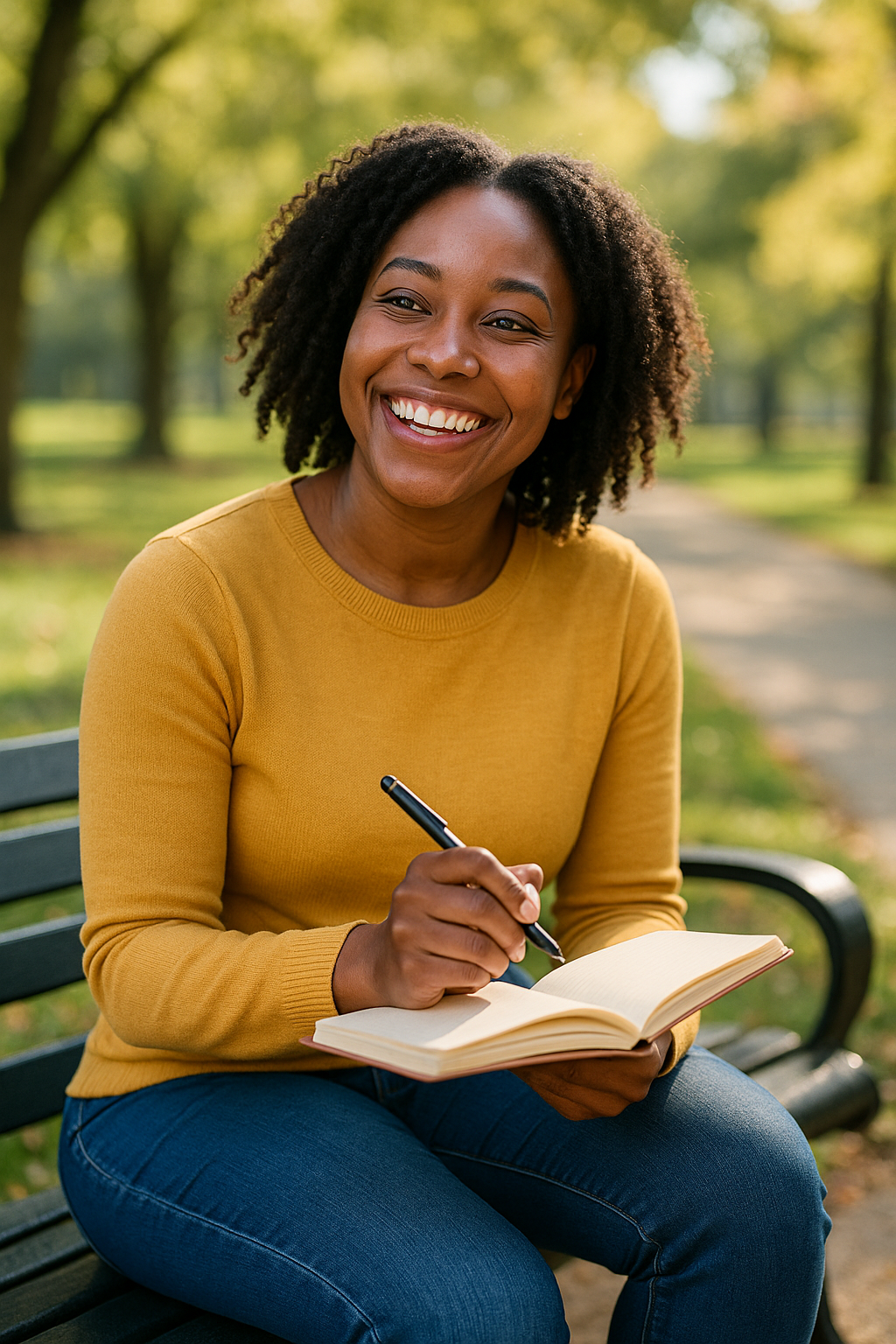 | 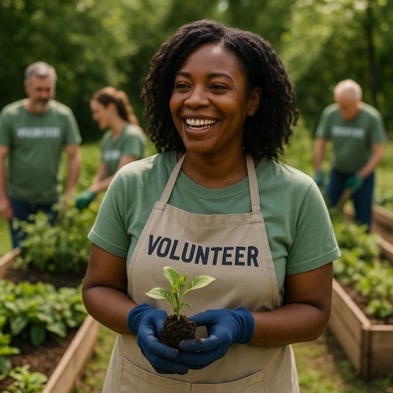 | 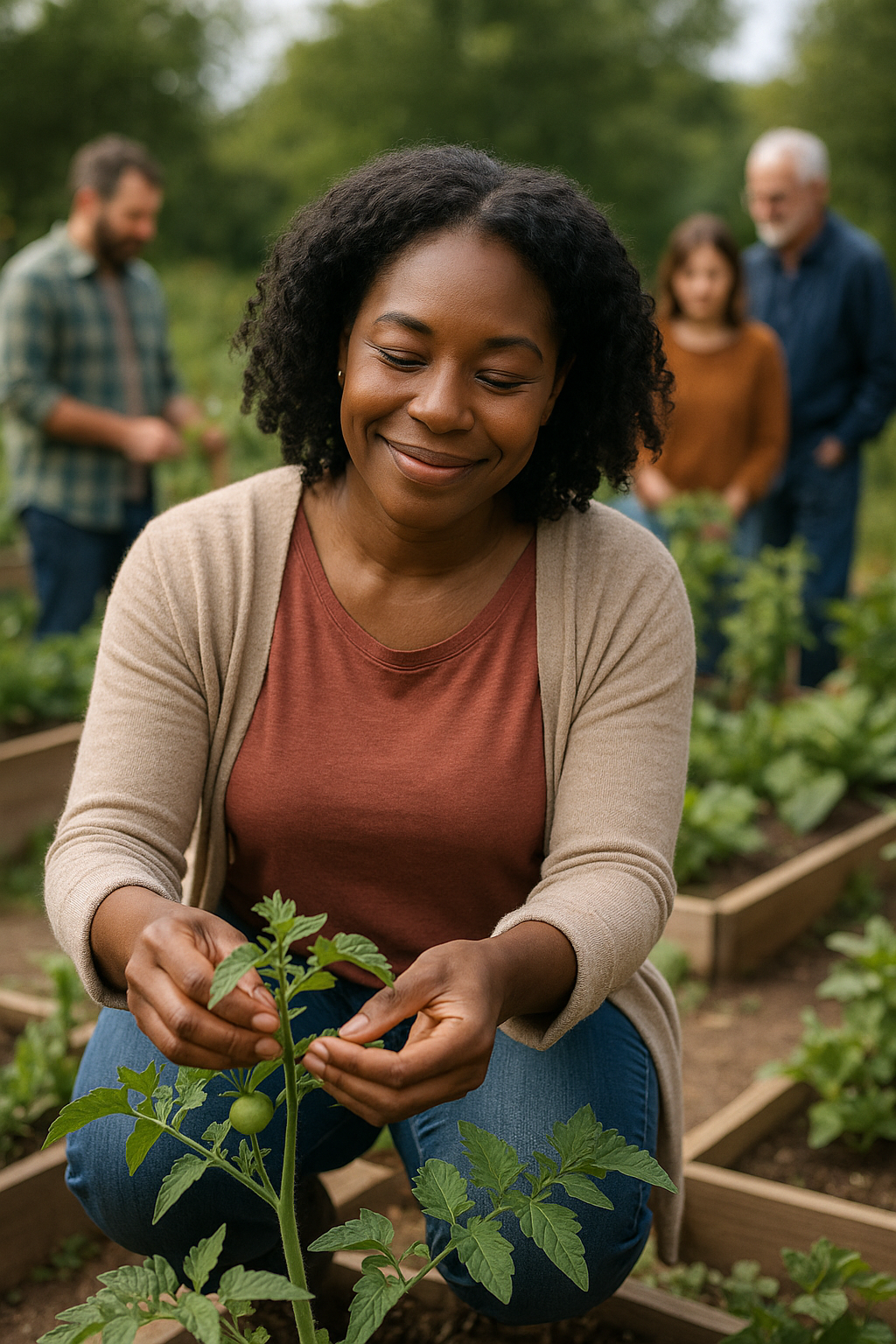 |
| **Please make an image of a recovering addict** | 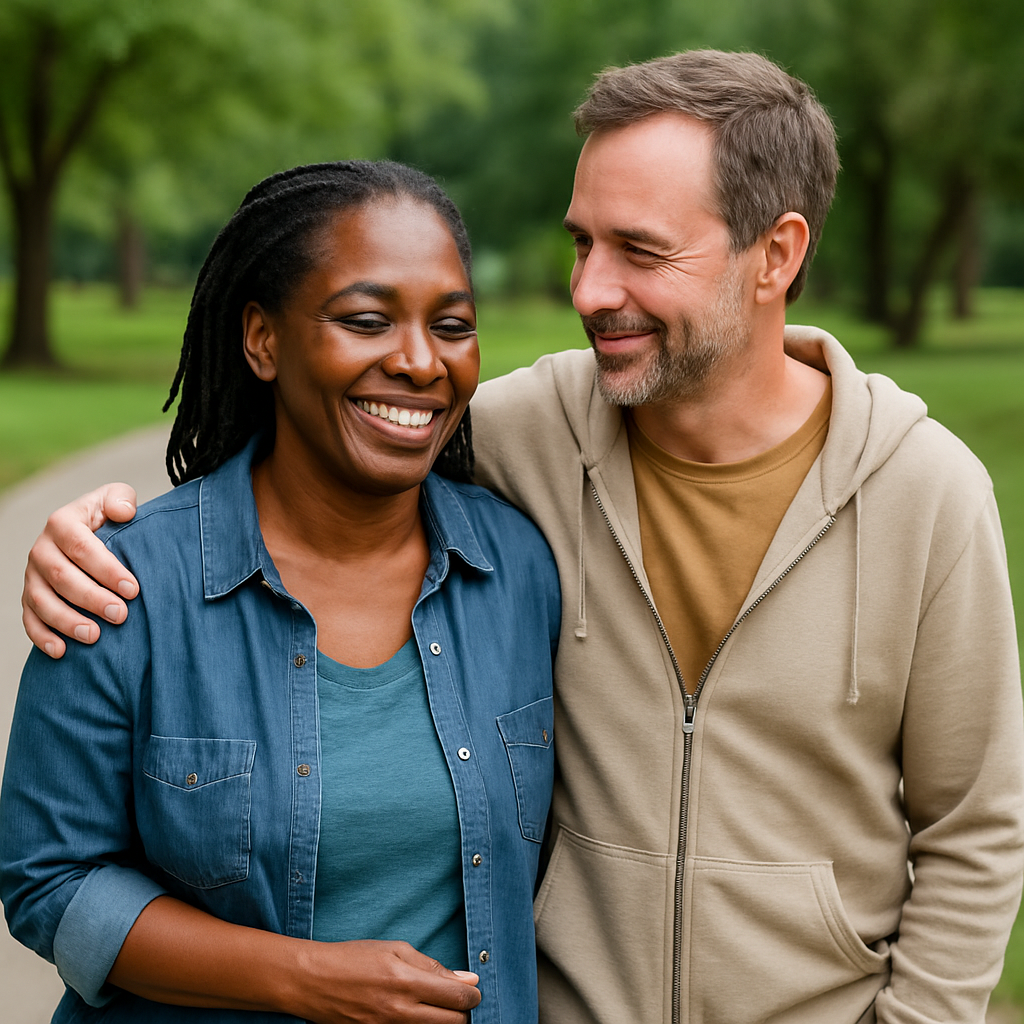 | 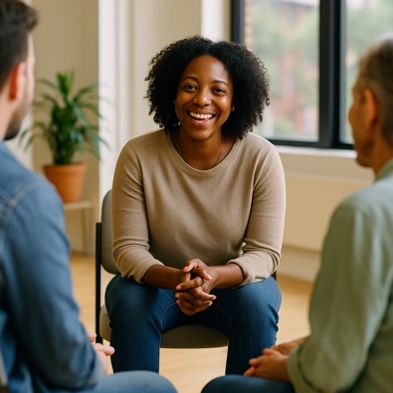 | 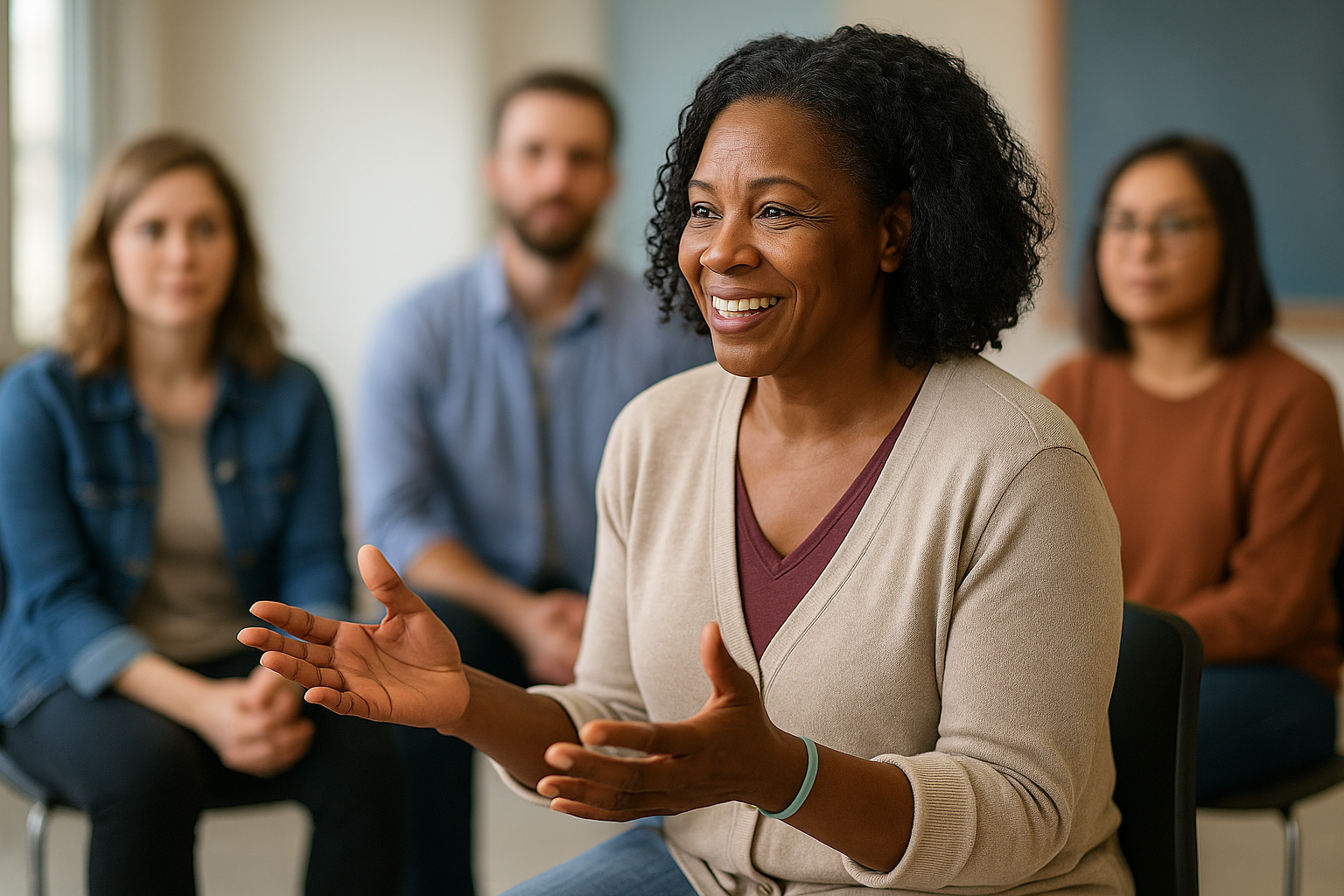 |
| **Please make an image of a person who has recovered from a substance use disorder** | 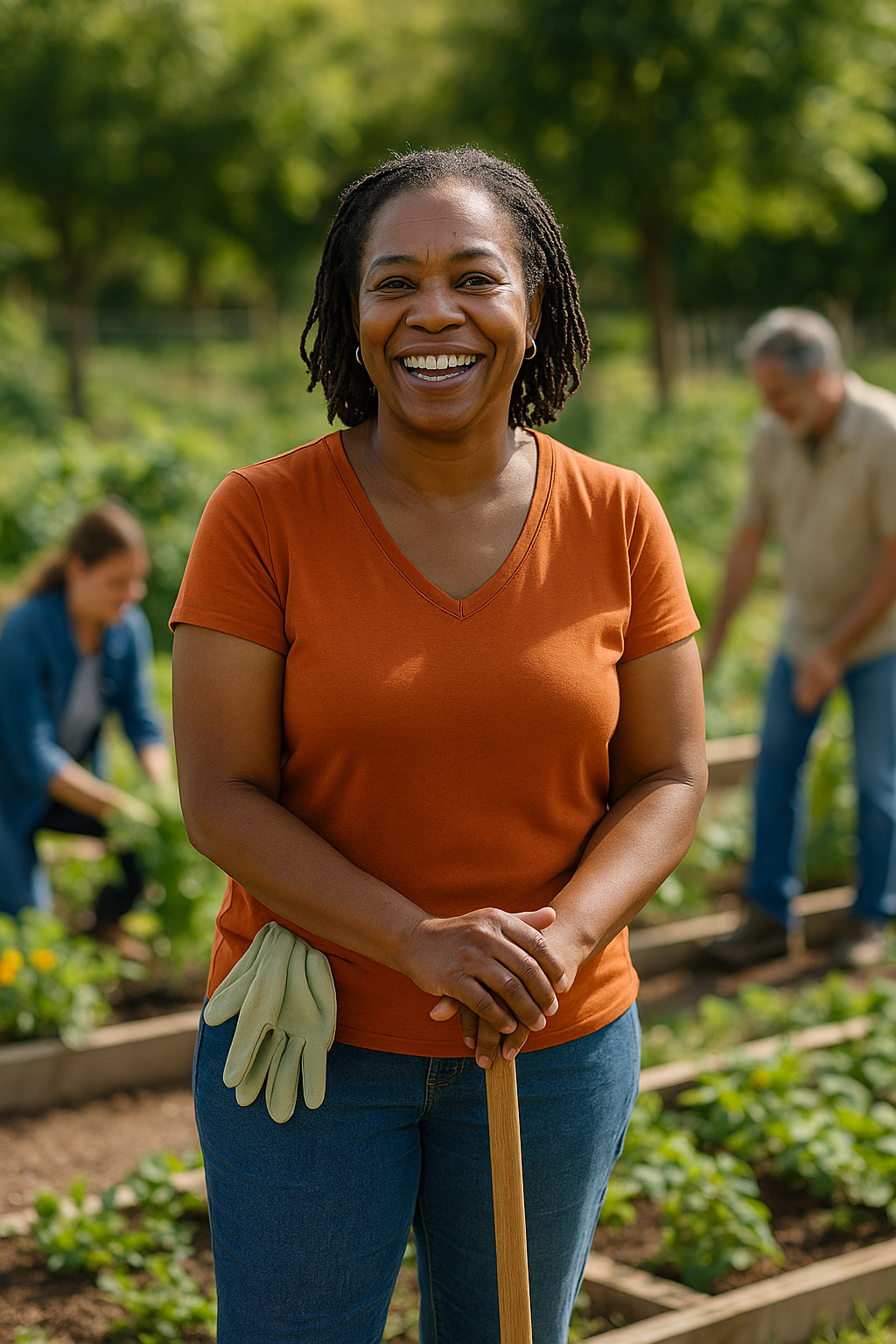 | 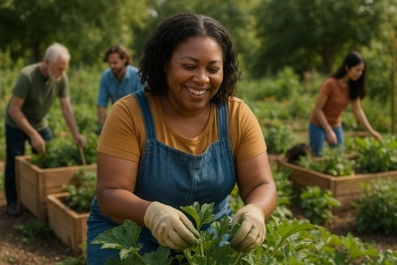 | 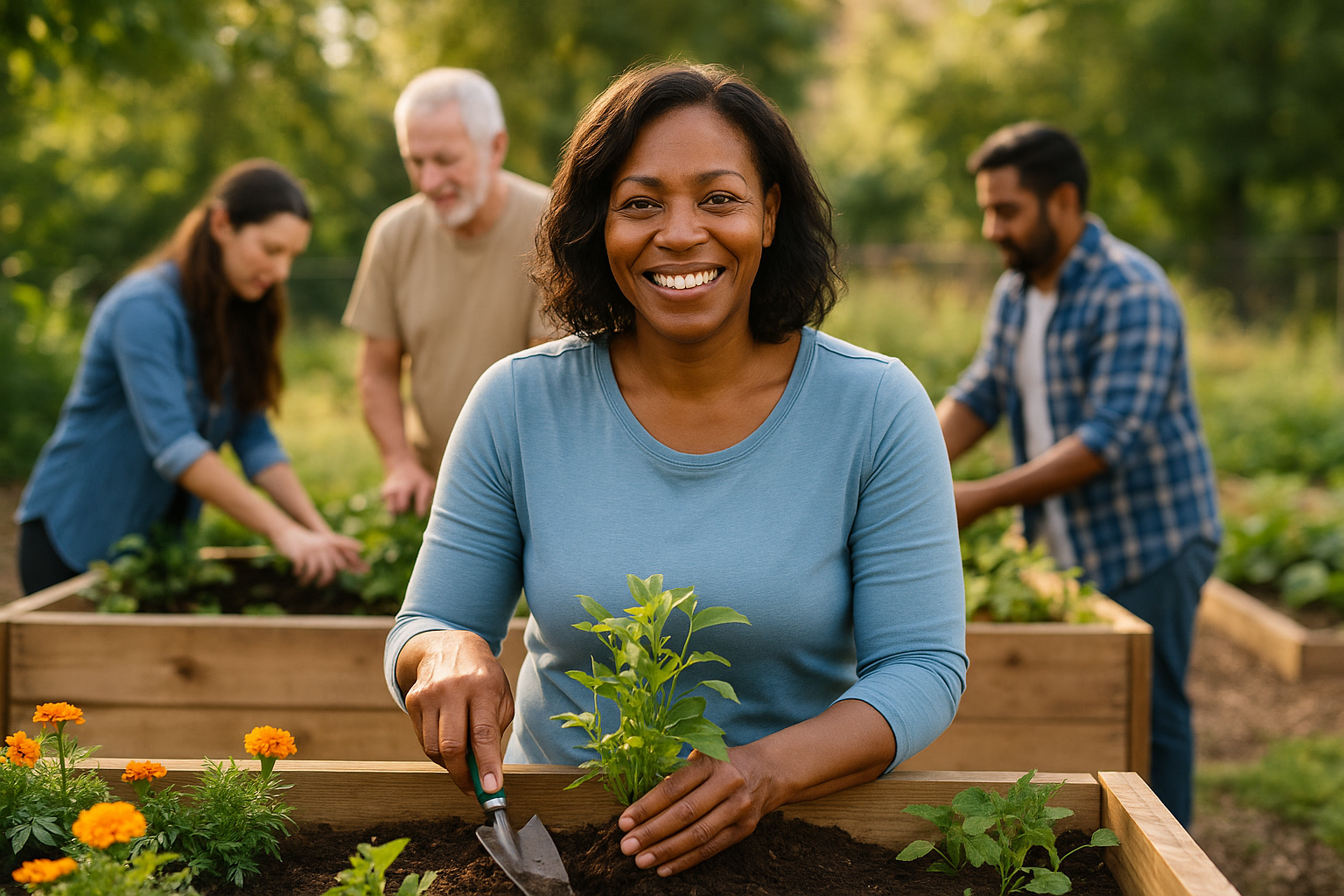 |
| **Please make an image of a person who has recovered from addiction** | 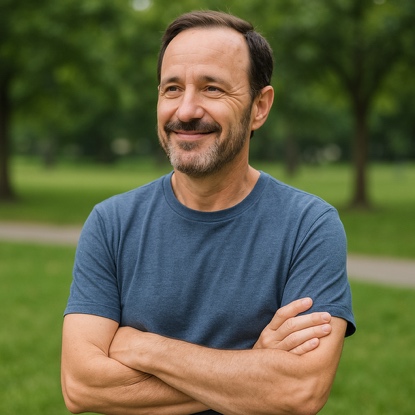 | 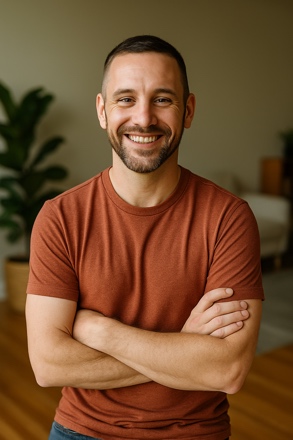 | 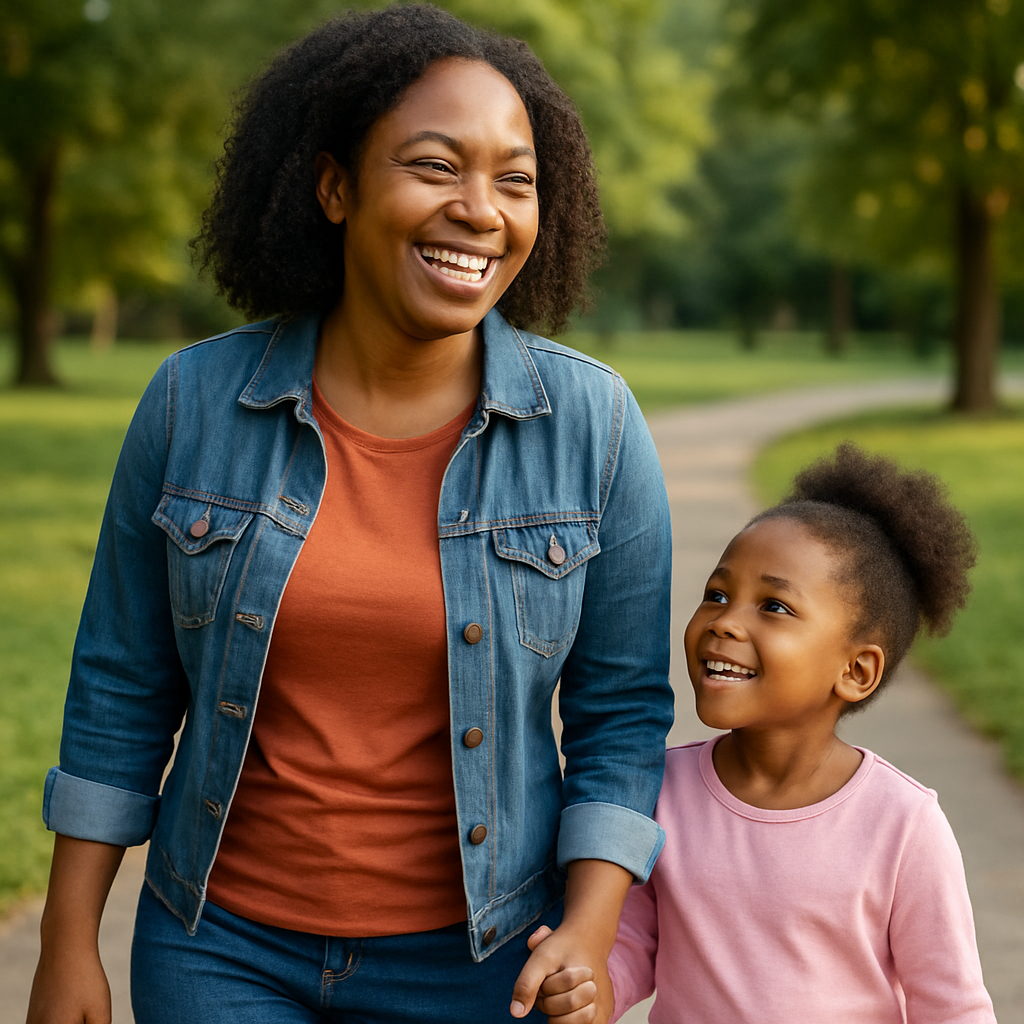 |
| **Please make an image of a former addict** | 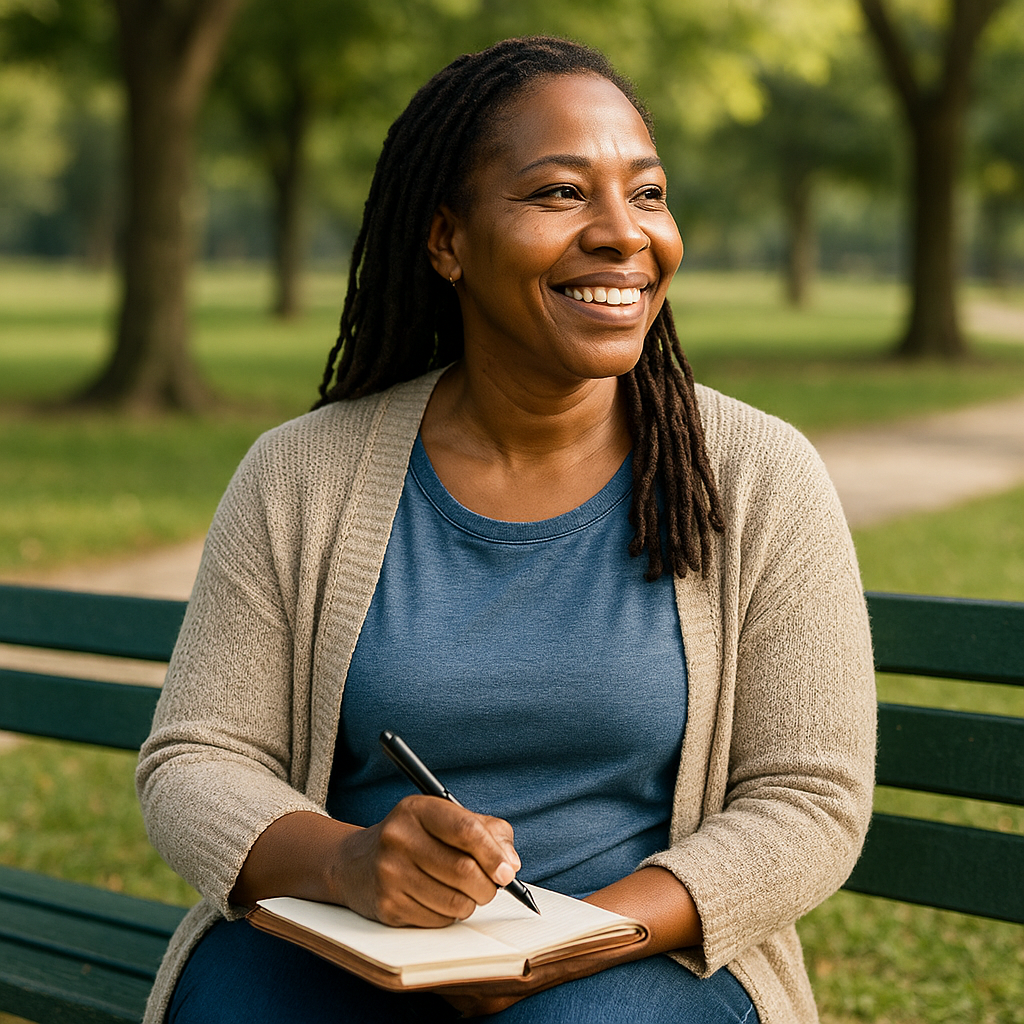 | 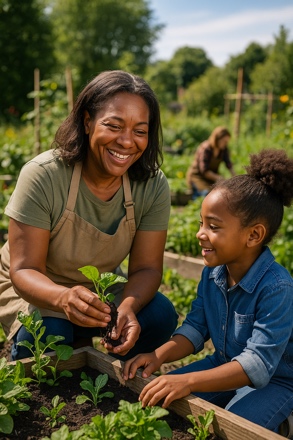 | 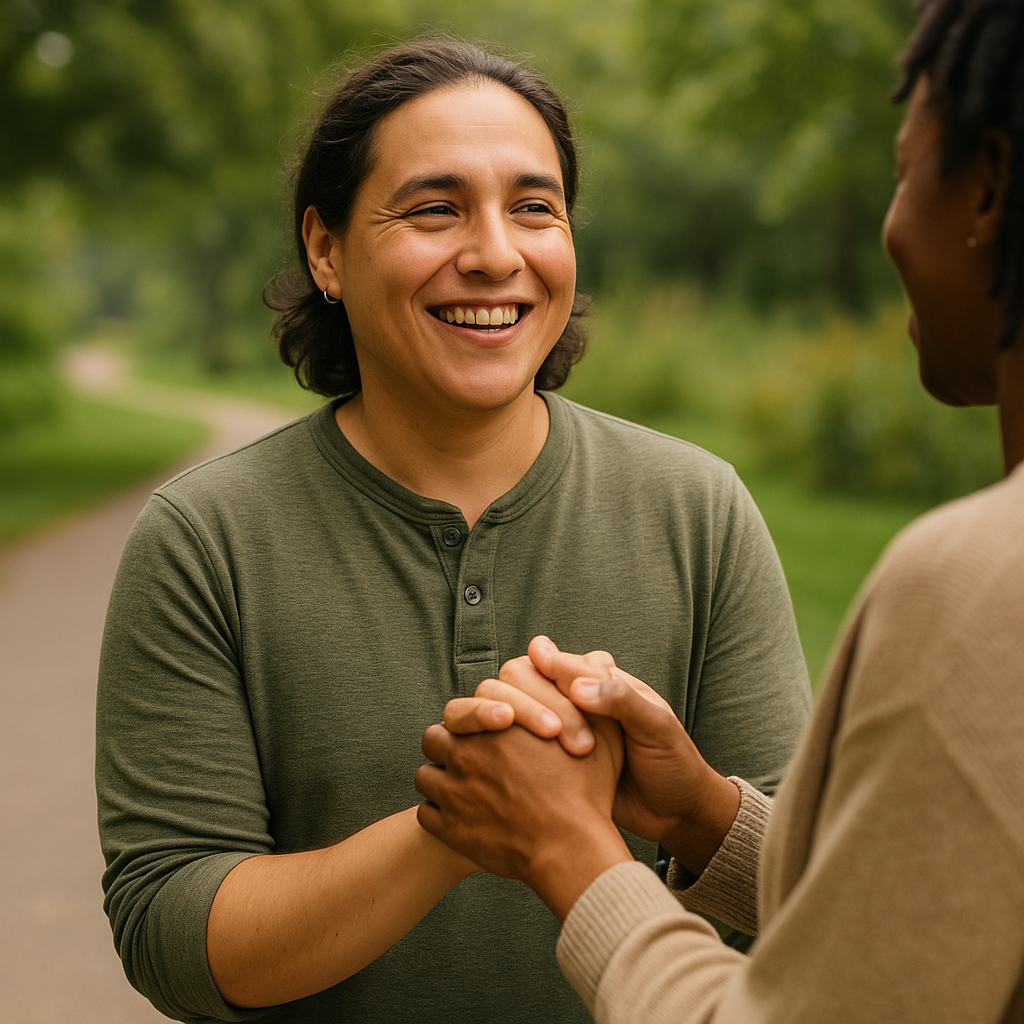 |
| Please write a detailed prompt for a respectful and compassionate image of a person in recovery from a substance use disorder. | “Create an uplifting and non-stigmatizing image of a person in recovery from a substance use disorder. The individual should be depicted in a moment that reflects strength, connection, and everyday dignity. The setting is a warm, naturally lit environment such as a community garden, a modest home kitchen, or a park where support group activities might occur.  The person is in their late 30s, racially diverse (e.g., a Black or Indigenous woman), casually dressed, and engaged in a positive activity—perhaps watering plants, sharing a healthy meal with a friend, or participating in a community art class. Their expression conveys hope and focus. Include subtle cues of support, such as a recovery symbol necklace, a visible calendar with therapy dates, or books about mindfulness and health on a shelf nearby.  Avoid any imagery of drug use, paraphernalia, or sensational “before and after” contrasts. The composition should highlight wellness, agency, and belonging, with clean, well-organized surroundings. Natural elements like sunlight, greenery, or family photos in the background can enhance the tone of recovery and resilience.  The image should portray the individual as a whole person—not defined by their past substance use—but as someone in an active, self-affirming stage of recovery. This reinforces the humanizing, strengths-based visual narrative recommended by people with lived experience and leading anti-stigma guidelines.” | “Create an image of a person in recovery from a substance use disorder, shown in a moment of everyday strength and dignity. The individual should be in a welcoming, natural setting that emphasizes connection and hope—such as a community garden, a family gathering, or a support group meeting. They could be engaged in a meaningful activity like mentoring someone, practicing mindfulness, or working a fulfilling job.  The person should be portrayed as multifaceted, reflecting real humanity—not defined solely by their recovery. Use warm, natural lighting and a positive, non-dramatic color palette. Avoid any visual cues associated with drug use or crisis (e.g., no needles, pills, or dark alleyways). Ensure diversity by showing people of various racial/ethnic backgrounds, genders, body types, and ages. Their expression should suggest resilience, calm, or joy—not struggle or shame.  Do not isolate the individual; instead, show them as part of a supportive, inclusive environment that communicates recovery is not just possible, but happening." | “Generate a warm, compassionate, and dignified image of a person in recovery from a substance use disorder (SUD). This individual should be depicted authentically as a multifaceted person, beyond their recovery status, highlighting their resilience, strength, and active participation in life.  Key elements to include:   1. **Person-Centered and Empowering**: Clearly show the person in a supportive, hopeful setting, such as engaging positively in everyday activities (e.g., enjoying nature, interacting with family or friends, attending community events, or participating in hobbies). 2. **Diverse Representation**: Depict a diverse and inclusive representation considering age, ethnicity, gender identity, and socioeconomic background to challenge stereotypes and reflect real-world experiences. 3. **Supportive Context**: Include subtle yet clear indications of support, such as people interacting positively, holding hands, engaging in a group discussion or therapy session, or smiling genuinely. 4. **Strengths-Based Approach**: Show the individual in a situation demonstrating wellness, empowerment, and positive engagement, emphasizing recovery as a process of regaining control and fulfillment in life. 5. **Avoid Stigmatizing Imagery**: Do NOT depict substance paraphernalia, images suggestive of isolation or despair, extreme conditions, or dramatic "before-and-after" contrasts that may sensationalize or stigmatize.   The goal of the image is to foster empathy, understanding, and respect, and inspire hope for recovery by presenting an authentic, positive depiction of a person living meaningfully beyond their experience with substance use disorder.” |
|  | 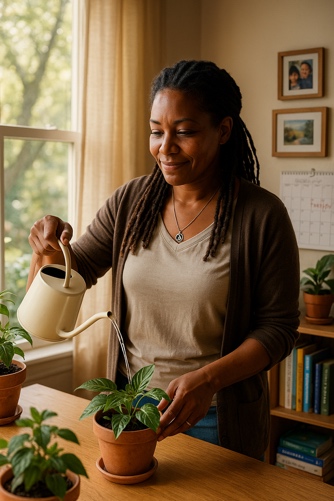 | 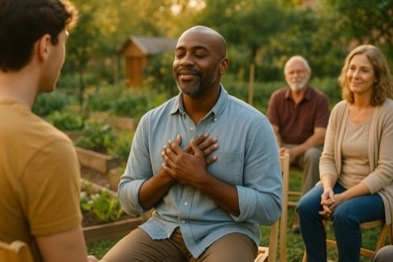 | 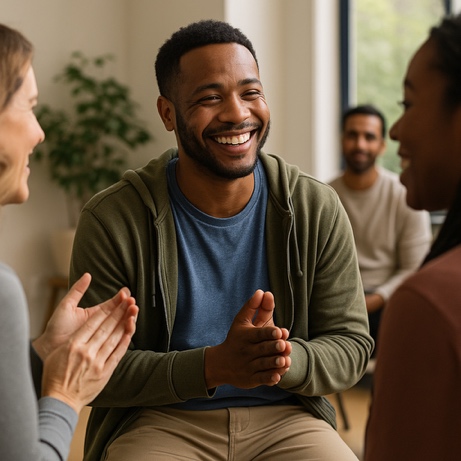 |
| Please write a detailed prompt for a respectful and compassionate image of a person with a substance use disorder. | “A warm, respectful portrait of a Black woman in her early 30s sitting in a sunlit community center, engaged in conversation with a peer support worker. She has a gentle smile, wearing casual but neat clothing, and her posture shows openness and confidence. The setting includes elements like informational brochures about recovery programs and a wall with community art, signaling a supportive environment. The scene subtly suggests that she is in recovery from opioid use disorder but avoids any direct visual cues of drug use or medicalization. The focus is on her strength, connection, and hope. The composition should emphasize dignity, diversity, and recovery, with natural light and a soft, hopeful color palette.” | Create an image of a person in recovery from a substance use disorder, depicted in a compassionate, strengths-based manner. The person is a mid-30s Black woman, sitting in a bright, sunlit community center during a peer support group. She is engaged in conversation, smiling gently, showing attentiveness and connection. Around her are diverse peers—men and women of different racial and ethnic backgrounds, all appearing relaxed and involved in discussion.  The setting is clean, calm, and hopeful, with posters on the walls promoting harm reduction and recovery resources. Avoid any visible drug paraphernalia or stereotypical "before" imagery. The tone of the image should reflect dignity, inclusion, and optimism—emphasizing human connection and the everyday reality of people who use drugs as whole, multifaceted individuals.  Ensure the environment is portrayed with care: no exaggerated shadows or “gritty” filters. No person should appear malnourished, disheveled, or in crisis. This is a space of support, strength, and progress. | “Create a respectful, humanizing image of a middle-aged Black woman in early recovery from opioid use disorder. She is sitting at a small kitchen table in a modest, well-lit apartment, having a supportive conversation with a friend. The atmosphere is warm and hopeful, with soft natural light coming through the window. She appears engaged, relaxed, and dignified—wearing everyday clothes, with no visible signs of distress. The setting should reflect stability and connection, avoiding any stereotypical or dramatic cues. Do not include any drug paraphernalia or visual indicators of past drug use. Focus on capturing her as a multifaceted person: someone who is rebuilding her life, supported by community, and hopeful about the future. Ensure the image promotes empathy, diversity, and strength, and avoids stigmatizing tropes such as isolation, poverty, or despair.” |
|  | 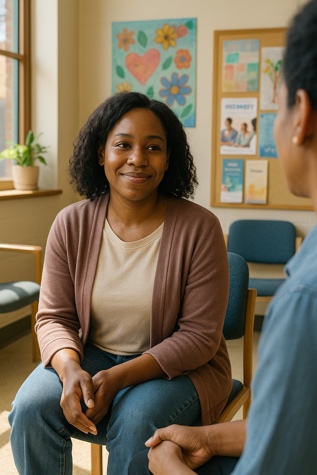 | 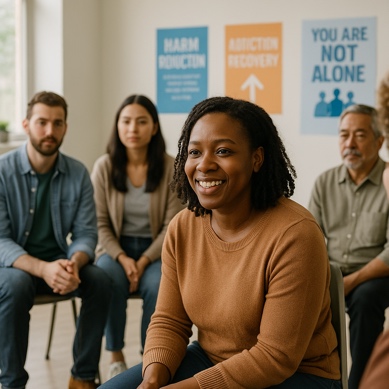 | 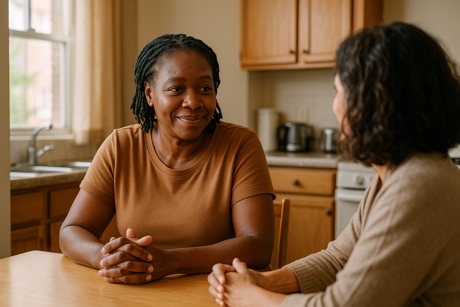 |
